# Sex- and Substance-Specific Associations of Circadian-Related Genes with Addiction in the UK Biobank Cohort Implicate Neuroplasticity Pathways

**DOI:** 10.3390/brainsci14121282

**Published:** 2024-12-20

**Authors:** Ayub Khan, Mete Minbay, Ziad Attia, Ahmet Ali Ay, Krista K. Ingram

**Affiliations:** 1 Department of Biology, Colgate University, Hamilton, NY 13346, USA; akhan@colgate.edu (A.K.); aay@colgate.edu (A.A.A.); 2 Department of Computer Science, Colgate University, Hamilton, NY 13346, USA; mminbay@colgate.edu (M.M.); zattia@colgate.edu (Z.A.); 3 Department of Mathematics, Colgate University, Hamilton, NY 13346, USA

**Keywords:** circadian clock, addiction, neuroplasticity, sex-specific associations, substance abuse

## Abstract

Background/Objectives: The circadian clockwork is implicated in the etiology of addiction, with circadian rhythm disruptions bidirectionally linked to substance abuse, but the molecular mechanisms that underlie this connection are not well known. Methods: Here, we use machine learning to reveal sex- and substance-specific associations with addiction in variants from 51 circadian-related genes (156,702 SNPs) in 98,800 participants from a UK Biobank cohort. We further analyze SNP associations in a subset of the cohort for substance-specific addictions (alcohol, illicit drugs (narcotics), and prescription drugs (opioids)). Results: We find robust (OR > 10) and novel sex-specific and substance-specific associations with variants in synaptic transcription factors (ZBTB20, CHRNB3) and hormone receptors (RORA), particularly in individuals addicted to narcotics and opioids. Circadian-related gene variants associated with male and female addiction were non-overlapping; variants in males primarily involve dopaminergic pathways, while variants in females factor in metabolic and inflammation pathways, with a novel gene association of female addiction with DELEC1, a gene of unknown function. Conclusions: Our findings underscore the complexity of genetic pathways associated with addiction, involving core clock genes and circadian-regulated pathways, and reveal novel circadian-related gene associations that will aid the development of targeted, sex-specific therapeutic interventions for substance abuse.

## 1. Introduction

Substance use disorders are influenced by multiple genetic and environmental factors and cause a complex interplay of neurobiological changes [1], which can be highly sex-specific in humans [2]. Recent work highlights the potentially significant role of the circadian clockwork in the etiology of addiction [3,4,5,6]. Although circadian genes are highly expressed in mood- and reward-related brain regions (reviewed in [7]) and likely mediate all steps of drug addiction in mice and humans [8,9,10,11], the precise molecular mechanisms of circadian influences on addiction are not yet well understood.

The endogenous circadian clock in the suprachiasmatic nucleus (SCN) controls the 24 h rhythms of the sleep–wake cycle and synchronizes the physiological cycling of peripheral clocks in tissues and organs via neurotransmitters and neuropeptides [12,13,14,15]. The timing of rhythmic clock gene expression among tissues and organs is variable among individuals and highly sex-dimorphic [16]. From both animal and human studies, a clear bidirectional relationship exists between disruptions in circadian rhythms and drug abuse. Circadian disruptions are common in individuals with substance use disorders, and drugs of abuse can, in turn, influence the synchronization of the SCN and circadian rhythms (reviewed in [11]).

Circadian misalignment and chronotype—individual preferences in the timing of the sleep–wake and activity cycles—tend to be risk factors for substance use, abuse, and relapse. The evening chronotype is associated with alcohol addiction, drug addiction, and addiction-like eating behavior [17,18,19] and is a major predictor of substance and alcohol dependence in young adults [20,21]. Night-shift workers, especially those with sleep disorders, also show a high prevalence of substance and alcohol dependencies [22,23]. In addition, circadian clock disruptions and related disorders (such as insomnia and depressive disorders) have high comorbidity with addiction [24] and are considered a major contributor in addiction relapse [25,26]. Thus, alterations in the circadian system may indirectly influence genetic and physiological pathways that increase the risk of addiction.

Circadian genes may also directly influence these physiological pathways via their roles as neurotransmitters and signaling molecules. For example, circadian gene variants regulating dopaminergic pathways can influence drug-induced rewards in the brain through management of energy supply to these pathways [27]. Polymorphisms in clock genes have been widely associated with drug and alcohol use disorders [28,29,30,31,32], and non-human studies also support a causal role of circadian rhythm dysfunctions in addiction [3,10,33], suggesting that circadian genes have both direct and indirect effects on pathways regulating addictive behaviors.

Although there is considerable support for the links between the circadian clock and addiction-related pathways, our understanding of the molecular clock mechanisms influencing sex-specific and substance-specific aspects of addiction remains limited. Hereafter, we use “circadian-related genes” to refer to the genes selected and analyzed in this study, including both core circadian genes and genes that are partially controlled by the circadian clock. The current study uses a comprehensive machine learning approach to elucidate the role of sex-specific variants of circadian-related genes in general addiction in a large population sample from the UK Biobank (UKB) cohort, hereafter Study One. Studies of UKB data have previously reported associations of addiction with sleep and mood disorders [34,35]. In this study, we analyzed 156,702 Single Nucleotide Polymorphisms (SNPs) from 51 genes in 98,800 participants (aged 39–72 years) within the UKB cohort. Additionally, we explored self-reported, substance-specific (alcohol, illicit drugs (e.g., narcotics), and OTC or prescription drugs (sedatives and painkillers, e.g., opioids)) addiction associated with circadian-related gene variants, hereafter Study Two. The genes surveyed in this study were selected by querying GeneCards with the terms “addiction” and “circadian”, supplemented by an extensive literature review. Here, we report novel circadian-related gene associations with addiction and highlight significant sex-specific and substance-specific genes and pathways that influence addiction risk in humans.

## 2. Materials and Methods

We followed a methodology similar to that of Minbay et al. [36]. We expand on those methods below, and a detailed description is provided in the Supplementary Materials. Supplementary Figure S1 provides an overview of the methods.

### 2.1. Data Collection

Our data were sourced from the UK Biobank (UKB), a database of genetic and health-related data (hereafter clinical factors) collected from more than 500,000 adults in the UK [37]. Genome-wide genotyping for all UKB participants was performed using the “Applied Biosystems UK BiLEVE Axiom Array” and the “UK Biobank Axiom Array”. Approximately 850,000 variants were directly measured, and over 90 million variants were imputed using the IMPUTE4 program and the Haplotype Reference Consortium, UK10K, and 1000 Genomes phase 3 reference panels. Quality control was conducted by testing for batch effects, plate effects, departures from Hardy–Weinberg equilibrium, sex effects, array effects, and discordance across control replicates. The socio-demographic and health-related factors for the participants were determined using electronic questionnaires [37]. We focused on participants who self-reported addiction or dependence on substances (excluding cigarettes and coffee) or behaviors like gambling (UKB data field 20401). We reviewed the literature and searched GeneCards for the terms “addiction” and “circadian” to identify the top 50 genes linked to circadian rhythm and/or addiction [38]. For instance, RORA was included as it is a core clock gene and has been associated with addiction in previous studies [39]. Similarly, ZBTB20 was included because it interacts with circadian genes, such as those involved in regulating evening behavioral activity [40], and it has been previously shown to be associated with nicotine addiction [41]. Additionally, one gene named DELEC1 was also included as it was significantly associated with mood disorders in the UKB cohort in a previous study from our lab [36]. The full gene list is provided in Supplementary Table S18. We then downloaded the corresponding imputed genotype and confounding clinical/demographic data (sex, age, chronotype, and Townsend Deprivation Index—TSDI) using UKB’s gfetch program. SNPs refer to all variants in the data (e.g., indels, point mutations, etc.) within these 51 genes or adjacent regions (±6 kbp). We excluded all participants who had missing or indeterminate (“prefer not to answer” or “do not know”) entries for any of the retrieved clinical factor fields, and additionally filtered participants who belonged to close kinship groups using the ukbb_parser Python library [42]. In Study One, 98,800 participants (42,501 males, 56,299 females), aged 39–72, remained after these filters. Of these, 5862 participants (3016 males, 2846 females) were marked as cases for addiction as per the self-reported data field.

For Study Two, we used a subset of 5366 participants (2749 males, 2617 females) who also self-reported addiction to alcohol (20,406), illicit/recreational drugs (20,456), and prescription/OTC medicine, including sedatives and painkillers (20,503)—parentheses indicate UKB data fields [37]. For simplicity, we use “narcotics” for illicit/recreational drugs and “opioids” for prescription/OTC medicine; these terms are examples and not fully representative of their categories. Of the 5366 participants, we marked 2246 (1262 males, 984 females) cases for alcohol addiction, 378 (227 males, 151 females) cases for narcotics addiction, and 804 (334 males, 470 females) cases for opioids addiction as per the respective self-reported data fields for each category.

SNP data were binarized according to the dominant model of inheritance, which compares the two less common genotypes (bb and Ab) to the common genotype (AA). Chronotype data were binarized by marking “definite evening types” as cases and the remaining three types as controls. Subsequent analyses were performed separately for male, female, and overall populations in both Study One and Study Two. Mediation and association rule learning (ARL) analyses were conducted only for Study One.

### 2.2. Feature Selection

We performed the feature selection on SNPs and epistatic interaction analysis on selected SNPs as detailed in the Supplementary Materials and Supplementary Figure S1. The final set of features included SNPs, SNP-SNP interactions, and confounding clinical/demographic factors.

### 2.3. Statistical Analysis

We used R for multivariate logistic regression on the final feature set. If the model did not converge, we eliminated collinearity recursively using Cramer’s V test (library: confintr) and GVIF scores (library: glmtoolbox) [43,44]. Variables with a Cramer’s V-score > 0.8 or GVIF > 5 were removed, and *p*-values were Benjamani–Hochberg-adjusted. Significant SNPs and SNP-SNP interactions (*p* < 0.05) were selected for further analyses. Risk SNPs were defined as those with OR > 1, and protective SNPs as those with OR < 1 in the final model, with corresponding genes determined as risk or protective. The regression results were visualized in networks using Gephi software Version 0.10.1 [45]. We used the mediation library in R to investigate the influence of chronotype on significant SNPs and SNP interactions from Study One, controlling for relevant covariates [46]. We used the arules package in R to perform ARL analysis of significant SNPs, epistatic SNP-SNP interactions, and chronotypes in Study One [47]. We restricted analysis to associations exhibiting a minimum lift value of 2 and a maximum factor group size limited to 5. Results were visualized using igraph and legendary packages [48,49].

### 2.4. Code Availability

The source code for all aforementioned procedures can be accessed at https://github.com/l1gh7vel/UK-Biobank_Addiction_Study.git (accessed on 7 June 2024).

## 3. Results

### 3.1. Study One: General Addiction

#### 3.1.1. Regression Analyses

**Figure 1:**
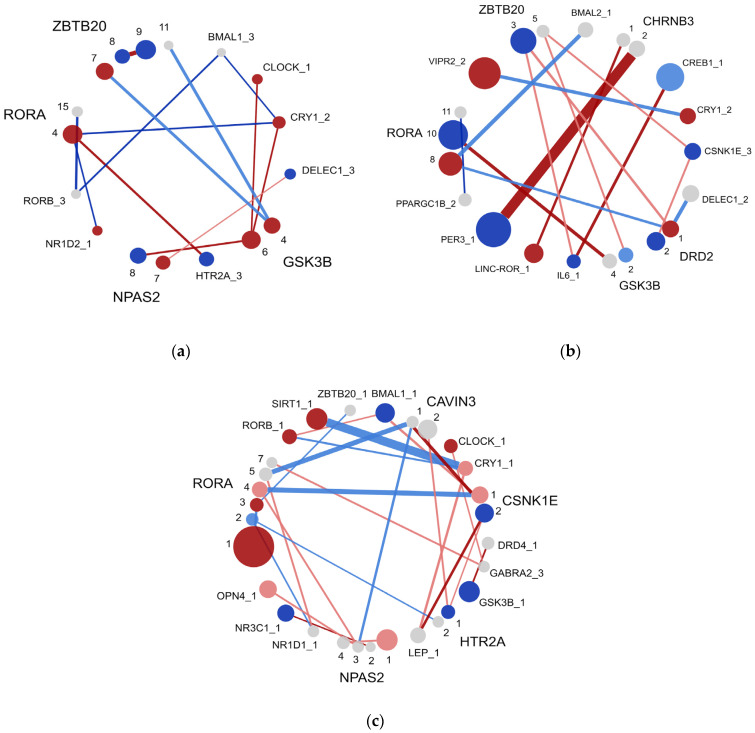
Associations of SNP-SNP epistatic interactions with addiction from overall, female, and male groups. Associations were calculated using logistic regression results in (**a**) overall, (**b**) female, and (**c**) male population samples, and were visualized using Gephi software. SNP nodes are represented by colored circles, where the node size is relative to odds ratios of the individual SNPs. Colors represent the risk categories of the variants according to the following key: gray = insignificant; light red = low risk; dark red = high risk; light blue = low protection; and dark blue = high protection. “Risk” indicates an odds ratio above 1, and “protection” refers to an odds ratio below 1. The “low” category indicates that the SNP’s association with addiction was not significant after *p* value correction, whereas “high” means the association remained significant after *p* value correction. Edges representing epistatic interactions are shown with the same color and size coding. Non-significant interactions (indicated by gray edges) were excluded. Mapping from the SNP label to rsIDs is given in Supplementary Table S5.

In Study One, we analyzed the associations of clock-related genes with addiction to any substance or behavior in the overall, male, and female samples. In the overall population, chronotype was associated with nearly twice the odds of addiction (OR 1.76, *p* < 0.001; Table 1). The same pattern for chronotype was seen in females and males (Table 1). For genetic factors, associations between clock-related gene variants and addiction in the overall group were weak (Table 1). The largest risk factor was a ZBTB20_ZBTB20 genotype (rs116191474_rs189085886; OR 3.89, *p* = 0.026; Table 1; Figure 1a).

More robust regression results were found for female and male samples. In females, the PER3_CHRNB3 pair increased the odds of addiction by more than eight times (OR 8.44, *p* < 0.01). Other significant variant pairs associated with addiction risk included GSK3B_RORA (OR 2.71, *p* = 0.046) and IL-6_CREB1 (OR 2.32, *p* = 0.021). Additionally, single SNPs in NPAS2 (OR 0.40, *p* < 0.01), RORB (OR 0.41, *p* = 0.023), and PER3 (OR 0.51, *p* = 0.012) were protective. A ZBTB20_ZBTB20 SNP pair was also protective (rs880744_rs114241174; OR 0.43, *p* = 0.028) (Table 1; Figure 1b). For males, a single SNP in RORA was a significant risk factor (OR 2.24, *p* < 0.01). Significant pairs included CAVIN3_CSNK1E (OR 3.00, *p* = 0.024), LEP_CSNK1E (OR 2.48, *p* = 0.023), and DRD4_GSK3B (OR 1.72, *p* = 0.018). No protective associations were observed at or above two times the odds in males (Table 1; Figure 1c).

#### 3.1.2. Association Rule Learning (ARL)

**Figure 2:**
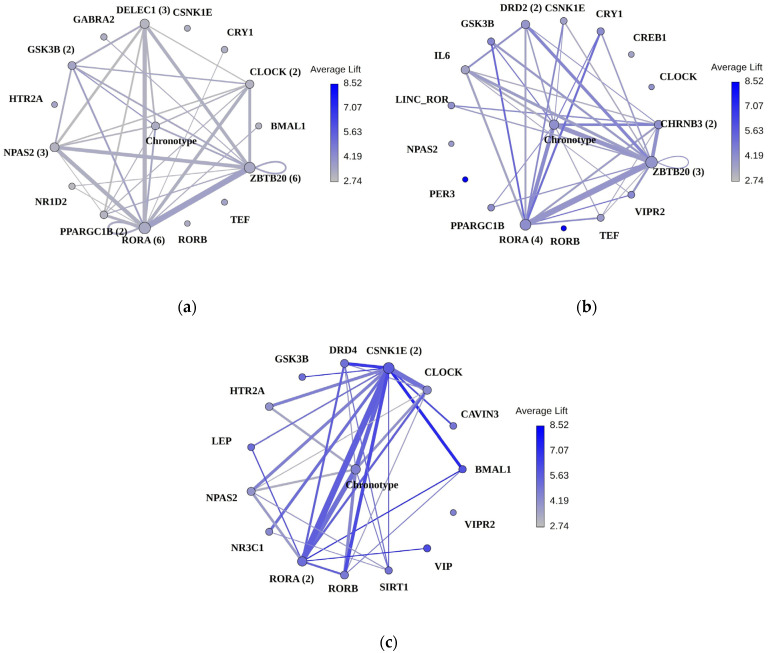
Association rule learning (ARL) networks. Networks show the co-occurrence of genes and chronotype in addiction risk “rules” within the UK Biobank population samples: (**a**) overall, (**b**) female, and (**c**) male. Genes or chronotype are represented as nodes, while the co-occurrence of these genes within rules is shown as edges connecting the nodes. The frequency of rule observations is reflected by the sizes of nodes and edges: larger nodes indicate items observed more frequently in rules, and larger edges signify pairs of items that commonly co-occur. The depth of node or edge color represents the average lift value of the rules containing that node or edge. Numbers in parentheses indicate the count of variants from a given gene that are present in the rules and contribute to the average lift. Lift serves as a measure of the increased likelihood of observing addiction in individuals whose genotype adheres to a specific rule, compared to a randomly selected individual.

Using ARL, the variants CLOCK, PPARGC1B, and DELEC1 co-occurred with the eveningness chronotype and showed the highest lift in the overall sample. The within-sex analysis revealed no overlapping variants and few overlapping genes between female and male samples. In the female group, a ZBTB20 variant co-occurred with variants from CHRNB3 and LINC_ROR and had the highest lift values, followed by the ZBTB20 and CHRNB3 variants combined with the eveningness chronotype. In males, a RORA variant—not observed in females—often combined with variants from CSNK1E, CLOCK, and NPAS2, as well as the eveningness chronotype for the highest lift values (Figure 2; Supplementary Tables S6–S8).

#### 3.1.3. Mediation Analysis

Associations of variants from NR1D2_RORA, HTR2A, ZBTB20, and BMAL1_RORB with addiction were partially mediated via chronotype in the overall population, and RORA and RORB showed partial chronotype mediation in females (Supplementary Table S4).

### 3.2. Study Two: Addiction Subtypes

#### 3.2.1. Alcohol Addiction

There were no significant genetic risk factors for alcohol addiction in the overall population that exceeded twice the odds, and within-sex associations were weak (Supplementary Tables S9–S11). In females, single variants in AANAT (OR 2.83, *p* < 0.01) and CHRNB3 (OR 1.94, *p* = 0.029) showed a significant risk for alcohol addiction. Other significant genetic risk factors included RORA_GSK3B (OR 2.85, *p* = 0.029). Protective factors included RORA (OR 0.46, *p* = 0.033), AANAT_RORA (OR 0.16, *p* = 0.042), and RORA_GSK3B (OR 0.32, *p* = 0.022) (Table 2). In males, factors exhibiting significant alcohol addiction risk included CHRNB3 (OR 2.35, *p* < 0.001), DRD4 (OR 1.84, *p* = 0.018), and CHRNB3_ZBTB20 (OR 3.19, *p* = 0.018). Protective factors included ZBTB20 (OR 0.42, *p* < 0.01), CHRNB3_RORA (OR 0.25, *p* = 0.031), and DRD4_RORA (OR 0.40, *p* = 0.031) (Table 3).

#### 3.2.2. Illicit or Recreational Drug Addiction

In the entire overall sample, statistically significant genetic risk factors for narcotic addiction included ZBTB20_OPN4 (OR 2.76, *p* = 0.035), DELEC1_RORA (OR 2.34, *p* = 0.049), and ZBTB20_PPARGC1B (OR 2.16, *p* = 0.035). Protective factors included ZBTB20 with almost three times the odds of protection (OR 0.35, *p* = 0.038), OPN4 (OR 0.42, *p* = 0.015), and RORA_PPARGC1B (OR 0.43, *p* = 0.028) (Supplementary Table S12).

The odds ratios for addiction risk and protection were more robust in sex-specific analyses (Supplementary Tables S13 and S14). In females, robust genetic risk factors for illicit drug addiction included VIPR2_ZBTB20 (OR 839.72, *p* < 0.01), DELEC1_BMAL1 (OR 34.18, *p* = 0.029), and CSNK1D_BMAL1 (OR 16.24, *p* = 0.032). Protective factors included ZBTB20 (OR 0.11, *p* = 0.026), RORA (OR 0.39, *p* < 0.001), and DELEC1 (OR 0.39, *p* = 0.029) (Table 2). In males, significant risk factors included the pairs RORA_ZBTB20 (OR 10.09, *p* = 0.028), ZBTB20_ZBTB20 (rs73224513_rs17628822; OR 8.85, *p* = 0.028), and RORA_ZBTB20 (OR 3.76, *p* = 0.027). Protective factors included SNPs from PER1 (OR 0.39, *p* = 0.024), and RORA (OR 0.43, *p* = 0.03) (Table 3).

#### 3.2.3. Prescription or Over-the-Counter (OTC) Medication Addiction

Significant genetic risk factors in the overall group included CSNK1E (OR 2.36, *p* < 0.001), DELEC1_DRD2 (OR 2.57, *p* = 0.037), and RORB_ZBTB20 (OR 2.37, *p* = 0.037). Protective factors included CHRNB3 (O.59, *p* = 0.028), CSNK1E_ZBTB20 (OR 0.50, *p* = 0.037), and CRY1_RORA (OR 0.56, *p* = 0.044) (Supplementary Table S15).

As with illicit drugs, the odds ratios for OTC medications were more robust in sex-specific analyses (Supplementary Tables S16 and S17). In females, robust genetic risk factors included the pairs SIRT1_RORA (OR 37.76, *p* = 0.022), PER2_NR1D2 (OR 19.27, *p* = 0.027), and CSNK1D_NPAS2 (OR 4.05, *p* = 0.021). Protective factors included single SNPs from the two period genes, PER1 (OR 0.37, *p* = 0.040) and PER2 (OR 0.34, *p* = 0.021) (Table 2). In males, significant risk factors included RORA_RORA (rs79360097_rs8027032; OR 8.27, *p* = 0.048), NFIL3_RORA (OR 7.31, *p* = 0.035), and DRD4_ZBTB20 (OR 6.09, *p* = 0.048). Protective factors included multiple SNPS from RORA (OR 0.17, *p* = 0.048; OR 0.42, *p* = 0.015; OR 0.57, *p* < 0.001) and one from RORB (OR 0.28, *p* = 0.015) (Table 3 and Supplementary Table S17).

## 4. Discussion

In this study, we examine the associations of variants in 51 genes related to both the circadian clock and addiction in the UK Biobank (UKB) cohort. Our results highlight a complex interplay of genetic pathways associated with addiction including core circadian clock genes, as well as multiple circadian-regulated physiological pathways [7,50,51]. Importantly, single SNPs in transcription factors involved in synapses (ZBTB20) and hormone receptors (RORA) are strongly associated with addiction, both alone and in epistatic combination with other SNPs. The majority of SNP association patterns show strong sex-specific differences in line with recent studies, underscoring the sex specificity of circadian influence on addiction [50,51,52,53,54]. In addition, we report novel gene associations specific to addictions to alcohol, narcotics, and opioid drugs in the UKB cohort, including variants in DELEC1 and ZBTB20 genes. Depending on the addiction type, 30–67% of individuals with the risk variant genotypes self-report addiction, a four- to ten-fold increase in addiction reports compared to the baseline of 8% in the full cohort (of all genotypes).

### 4.1. Multiple Circadian-Regulated Molecular Pathways Are Associated with Addiction

We find multiple variants from several circadian-related genes associated with addiction, including PER3, ZBTB20, RORA & RORB, CSNK1E, NPAS2, AANAT, SIRT1, and CAVIN3. These clock genes have been previously associated with some form of addictive behavior in human or non-human models [39,55,56,57,58,59]. For example, PER3 variations have been linked to stress and addiction in mice, as well as heroin dependence in a Han Chinese population [60,61]. We also report gene variants from the following circadian-influenced pathways associated with addiction: inflammation (IL6), synaptic plasticity (GSK3B, CREB1, CHRNB3), monoamine pathways (DRD4, HTR2A), and metabolic/stress pathways (GSK3B, PPARGC1B, VIPR1). These genes and/or pathways have been previously linked to addiction in prior studies [62,63,64]. Interestingly, the effects of these circadian-related genes on addiction were found to be only partially and weakly mediated by the eveningness chronotype. Given the strong associations between chronotype and addiction behavior in previous studies, we expected that associations of circadian variants with addiction may be partially or fully mediated by chronotype. Our mediation analyses found variants in NR1D2_RORA, HTR2A, ZBTB20, and BMAL1_RORB were partially mediated via chronotype in the overall population, and RORA and RORB showed partial chronotype mediation in females. Previous studies have found variants in NR1D2, RORA, RORB, and HTR2A associated with chronotype [65,66,67,68,69,70], although the specific variants reported were not significant for addiction risk in this study. Our mediation results suggest potential indirect effects of these genes on addiction, with altered circadian phase a likely mechanism for the modulation of downstream physiological or neuroplasticity pathways. Although circadian pathways are highly represented in association with addiction, and chronotype was a significant risk factor for addiction in all three groups, most of the epistatic effects in this study are likely directly regulated by the circadian genes rather than via disrupted rhythms. Thus, the effects of some circadian genes on addiction may not depend on the altered circadian phases that dictate chronotype.

The results from Studies One and Two highlight two significant novel gene associations that deserve additional consideration in future translational research on addiction. We report the robust association of two DELEC1 variants (rs41278693 and rs2992140) with illicit drug addiction in females. While the role of DELEC1 in circadian rhythms or substance abuse remains uncertain, it may contribute to tumor suppression and has been implicated in female depression in a recent study [36]. Similarly, ZBTB20, a transcription factor involved in synapses and neurodevelopmental disorders [71], has not been previously linked to addiction, although it did appear in a GWAS study on smoking initiation [72]. This gene is particularly interesting because it was the only significant gene reported in a GWAS for seasonal affective disorder (SAD) [73]. Two recent studies have reported ZBTB20 variants associated with both major depressive disorder and SAD in a sex-specific manner [36,74]. Additional studies are required to better understand the sex-specific functions of DELEC1 and ZBTB20 in depression and addiction.

### 4.2. Epistatic Interactions of Synaptic Molecules Strongly Associated with Addiction

Recent whole-genome studies of addiction report hundreds of genes influencing substance abuse and confirm the polygenic nature of addiction risk, resulting from many variants with small effects [75,76]. The benefit of using machine learning techniques in the current study is the ability to consider both single variants and possible interactions between variants, or between variants and clinical/demographic factors, to better understand the genetic basis of addiction. Our two-SNP interactions yielded the greatest odds of addiction risk or protection in our logistic regression results, and the ARL rules exhibited high lift values, supporting the importance of epistatic interactions. Importantly, the most robust epistatic interactions included one of four genes encoding either synaptic molecules or hormone receptors. For instance, an SNP-SNP interaction pair of PER3 and CHRNB3 was the strongest risk factor for females for general addiction, and an SNP from SIRT1 in combination with CRY1 was the strongest protective factor for the male population. CHRNB3/A6 synaptic transmission genes have been linked to nicotine and cocaine dependencies [77,78,79] and SIRT1 is an NAD-dependent histone deacetylase involved in circadian gene regulation and linked to sensitization to heroin [80,81]. The highest variants of influence across all groups are polymorphisms found in RORA, a circadian-related hormone receptor, and in ZBTB20. Our results thus align with the literature highlighting the critical influence of circadian interactions with reward centers in the brain on the neurobiology of addiction.

### 4.3. Sex Specificity in Genetic Pathways Influencing Both General and Specific Addiction

Addiction manifests differently in males and females [82,83], but genetic studies of the molecular mechanisms underlying these sex differences are still nascent, especially in humans [2,84,85]. This study highlights genetic differences that may explain some of these sex-specific patterns. Notably, we found strong associations with circadian and synaptic pathways in both males and females, but relatively non-overlapping downstream physiological pathways. Female gene associations involved primarily metabolic and inflammation pathways, while male gene associations had stronger effects from genetic variants in dopaminergic pathways.

In females, genes linked to metabolism like GSK3B, RORA, RORB, CREB1, and, importantly, vasoactive intestinal peptide receptor 2 (VIPR2), are implicated in multiple types of addiction in the UKB female cohort. VIPR2, which was strongly associated with narcotic drug addiction in females, has been associated with opioid addiction and cocaine dependence in previous studies [57,86]. A variant in DELEC1 also appears in a robust association with addiction to narcotics in females in our study, but as noted above, the function of this gene and this variant are yet unknown. Circadian genes, including the period genes ZBTB20, PER2, PER3, SIRT1, BMAL1, NR1D2, and CSNK1D, and the melatonin production gene, AANAT, also show strong associations with female addiction. Essential clock gene polymorphisms in PER2, BMAL1, NR1D2, SIRT1, and AANAT have been associated with alcohol consumption and dependency in mouse and human studies [10,87,88,89]. Similarly, NR1D2, BMAL1, and CSNK1D rhythms were disrupted in opioid use disorder (OUD) and AUD patients compared to controls [90,91].

In males, gene variants associated with neurogenesis and synaptic plasticity, such as CHRNB3 and NFIL3, a gene involved in PER1 and PER2 repression, were significant in our analyses. NFIL3 has been shown to be deregulated in cannabidiol consumption in mice microglial cells [39]. Multiple variants of the monoamine gene DRD4, as well as variants from metabolic genes Leptin (LEP) and GSK3B, also played a crucial role in addiction in males. In the previous literature, DRD4 was associated sex-specifically with alcohol consumption physiopathology [92]. Leptin has also been associated with addictions in prior studies, as its concentration in blood was associated with higher alcohol craving [93] and may have sex-specific effects [94,95]. Robustly associated circadian genes for males include ZBTB20 and RORA, as described above, and CSNK1E. CSNK1E has been nominally linked to heroin addiction in humans [96] and sensitivity to psychostimulants and opioids (methamphetamine and fentanyl) in a previous mice study [97].

Our results suggest potential circadian-related pathways that may influence sex-specific addiction risk. The effect of circadian genes on dopaminergic pathways in males may indicate that susceptibility in addiction risk is strongly modulated by reward systems. For females, the connections may be less direct, with the effects of circadian genes on inflammation and metabolic pathways in females altering the function of the hypothalamic–pituitary–adrenal (HPA) axis and glucocorticoid systems. Altering function in the HPA axis and/or glucocorticoid pathways may affect reward mechanisms in females via multiple neuroendocrine pathways, including sex-specific estradiol-regulated pathways [83]. Although the precise mechanisms are not yet clear, our findings suggest that treatments for substance abuse may benefit from sex-specific therapeutic treatments and behavioral approaches.

### 4.4. Dissection of Addiction Patterns Reveals Distinct Pathways for Substance Abuse

We found no robust relationships between alcohol addiction and genetic factors across the cohort groups in Study Two. However, multiple CHRNB3 gene variants showed small but significant associations with alcohol addiction, suggesting that synaptic transmission pathways may play a role in alcohol addiction. In comparison to the analyses for narcotic and opioid addictions, our results suggest that circadian-related genetic factors have lower odds ratios, and thus smaller effect sizes, for alcohol addiction than for other substance use disorders in humans. We speculate that differences in how the circadian clockwork interacts with neuroplasticity or reward pathways in narcotics and opioid addiction may explain the stronger associations of circadian-related genes with these addiction types, but more research is needed to understand these associations.

Gene associations with illicit drug addiction (narcotics) and prescription drug addiction (opioids) are more robust when analyzed separately by sex. In females, individuals reporting narcotic addiction show robust associations with circadian clock genes (ZBTB20, RORA, BMAL, and CSNK1D), metabolic processes (VIPR), and DELEC1. In males, there is a strong association between synaptic transmission and circadian rhythms (RORA and ZBTB20). Similarly, females reporting prescription or opioid addictions show robust associations with core clock genes (PER2, NR1D2, RORA, and SIRT1) indicating a clock-mediated process underlying opioid addiction. In males, variants in genes related to synaptic transmission and circadian rhythms (RORA and ZBTB20) and dopamine transmission (DRD4) elevate addiction risk. Overall, our results suggest sex specificity in the pathways involved in all three addiction types, with particularly robust sex differences in variants associated with narcotic and opioid addiction.

### 4.5. Limitations

Limitations of our study include the use of self-reported addiction data from the UKB and our focus on genes related to the circadian clock, as the analyses will not capture additional physiological factors influencing addiction. In addition, our use of strict diagnostic criteria limited the sample size from the set of individuals who might report substance abuse problems. However, these criteria allow for critical, clinically relevant cohort samples for robust gene association analyses. Due to these strict criteria, we altered the minimum threshold for frequency of SNPs by dependency type in order to optimize testing without losing rare but potentially valuable SNPs, particularly in the sex-stratified samples. For some SNPs, this convention, along with the small sample sizes, resulted in large confidence intervals surrounding the odds ratio values. Our study cohort includes only the limited age range represented by the adult participants in the UKB (39–72 years). Although people of all ages are impacted by substance use disorders, the limited age range of this study may make it challenging to generalize the genetic associations of adult addiction to adolescents or young adult populations. We did not consider the effects of circadian light hygiene or patterns of physical activity on addiction risk, although these factors could be significant predictors of psychological disorders [98,99,100]. Finally, the use of the UKB data cohort, which is composed primarily of Caucasian individuals of European descent, may limit the generalization of our results to individuals of other ethnicities.

## 5. Conclusions

In conclusion, a systems approach utilizing a large database was selected in this study to investigate how circadian clock regulation might influence the risk of addictive behaviors. The findings emphasize the intricate nature of physiological pathways associated with addiction and underscore the distinct, non-overlapping genetic associations observed between males and females. Circadian rhythm regulation of addictive behaviors via individual gene variants or two-variant combinations is implicated in two independent statistical analyses, with circadian signaling and synaptic genes robustly associated with addiction. Metabolic and inflammatory pathways are linked to female addiction, and dopaminergic-related gene pathways are linked to male addiction. Although circadian-related pathways are robust in these analyses, few of the associations are significantly mediated by chronotype, suggesting direct effects of circadian genes on addiction pathways. Overall, our findings indicate that circadian influences on addiction operate through distinct pathways that are sex- and substance-specific. Thus, treatments for all three types of substance abuse may benefit from sex-specific therapeutic treatments and behavioral approaches.

## Figures and Tables

**Table 1:** Circadian gene polymorphisms and clinical factors associated with addiction in the overall, female, and male sample populations. Significant addiction risk and protective factors in the overall UK Biobank population sample were identified using multivariate logistic regression (adjusted *p* < 0.05). Significant results are shown with odds ratios (ORs) and 95% confidence intervals (CIs).

	Variable	rs #	OR [95% CI]	*p* Value (adj)
Overall				
Risk	ZBTB20_ZBTB20	rs116191474_rs189085886	3.89 [1.12–10.43]	0.014 (0.026)
	HTR2A_RORA	rs80298007_rs551091515	1.97 [1.13–3.27]	0.012 (0.024)
	Chronotype		1.76 [1.63–1.90]	<0.001 (<0.001)
Protective	ZBTB20_PPARGC1B	rs574306550_rs17110463	0.48 [0.28–0.78]	<0.01 (0.017)
	RORA_RORB	rs28410611_rs11144030	0.49 [0.24–0.90]	0.033 (0.047)
Female				
Risk	PER3_CHRNB3	rs143075778_rs56339363	8.44 [2.53–24.25]	<0.001 (<0.01)
	GSK3B_RORA	rs575769812_rs28652652	2.71 [1.18–5.81]	0.014 (0.046)
	IL6_CREB1	rs73683966_rs76863021	2.32 [1.31–4.16]	<0.01 (0.021)
	Chronotype		1.62 [1.45–1.81]	<0.001 (<0.001)
Protective	NPAS2	rs150840995	0.40 [0.23–0.64]	<0.001 (<0.01)
	RORB	rs188874518	0.41 [0.21–0.73]	<0.01 (0.023)
	ZBTB20_ZBTB20	rs880744_rs114241174	0.43 [0.23–0.77]	<0.01 (0.028)
	PER3	rs143075778	0.51 [0.33–0.76]	<0.01 (0.012)
Male				
Risk	CAVIN3_CSNK1E	rs112063177_rs138711638	3.00 [1.33–6.25]	<0.01 (0.024)
	LEP_CSNK1E	rs77947631_rs113075284	2.48 [1.29–4.52]	<0.01 (0.023)
	RORA	rs147861260	2.24 [1.54–3.17]	<0.001 (<0.01)
	Chronotype		1.80 [1.62–2.00]	<0.001 (<0.001)
	DRD4_GSK3B	rs79177795_rs13082848	1.72 [1.19–2.44]	<0.01 (0.018)

**Table 2:** Circadian gene polymorphism associated with addiction subtypes in the female sample population. Significant risk and protective factors for addictions to alcohol, illicit or recreational drugs, and prescription or over-the-counter medications in the UK Biobank female population were found using multivariate logistic regression (adjusted *p* < 0.05). Factors include SNPs or SNP-SNP epistatic interactions. Significant results are shown with odds ratios (ORs) and 95% confidence intervals (CIs). Clinical factors are not included.

	Variable	rs #	OR [95% CI]	*p* Value (adj)
Alcohol				
Risk	RORA_GSK3B	rs75181035_ch3:119713907	2.85 [1.40–5.88]	<0.01 (0.029)
	AANAT	rs113263038	2.83 [1.74–4.68]	<0.001 (<0.01)
	CHRNB3	rs138834080	1.94 [1.24–3.04]	<0.01 (0.029)
Protective	AANAT_RORA	rs113263038_rs75181035	0.16 [0.03–0.59]	0.011 (0.042)
	RORA_GSK3B	rs60257905_ch3:119713907	0.32 [0.15–0.66]	<0.01 (0.022)
	RORA	rs8024334	0.46 [0.26–0.79]	<0.01 (0.033)
Illicit or Recreational Drugs				
Risk	VIPR2_ZBTB20	rs183433583_rs77359140	839.72 * [19.81–40471]	<0.001 (<0.01)
	DELEC1_BMAL1	rs41278693_rs34188368	34.18 [3.16–841.14]	<0.01 (0.029)
	CSNK1D_BMAL1	rs76390553_rs34188368	16.24 [2.06–149.24]	<0.01 (0.032)
Protective	ZBTB20	rs77359140	0.11 [0.02–0.42]	<0.01 (0.026)
	RORA	ch15:61377024	0.39 [0.24–0.61]	<0.001 (<0.001)
	DELEC1	rs2992140	0.39 [0.19–0.75]	<0.01 (0.029)
Prescription or Over-the-Counter Medication				
Risk	SIRT1_RORA	rs113693349_rs8042801	37.76 [3.82–623.79]	<0.01 (0.022)
	PER2_NR1D2	rs62194938_rs149810501	19.27 [2.39–184.23]	<0.01 (0.027)
	CSNK1D_NPAS2	rs117549365_rs12712082	4.05 [1.61–10.45]	<0.01 (0.021)
Protective	PER2	rs62194938	0.34 [0.16–0.65]	<0.01 (0.021)
	PER1	rs3027191	0.37 [0.16–0.75]	0.011 (0.040)

* This SNP has an inflated odds ratio due to the small sample size: only 4 individuals had this variant, one of whom had an addiction to narcotics, which is much higher than the 6% prevalence of addiction in the rest of the narcotics group.

**Table 3:** Circadian gene polymorphism associated with addiction subtypes in the male sample population. Significant risk and protective factors for addictions to alcohol, illicit or recreational drugs, and prescription or over-the-counter medications in the UK Biobank male population were found using multivariate logistic regression (adjusted *p* < 0.05). Factors include SNPs, or SNP-SNP epistatic interactions. Significant results are shown with odds ratios (ORs) and 95% confidence intervals (CIs). Clinical factors are not included.

	Variable	rs #	OR [95% CI]	*p* Value (adj)
Alcohol				
Risk	CHRNB3_ZBTB20	rs55828312_rs113104147	3.19 [1.54–6.72]	<0.01 (0.018)
	CHRNB3	rs41272375	2.35 [1.62–3.46]	<0.001 (<0.001)
	DRD4	rs916455	1.84 [1.25–2.73]	<0.01 (0.018)
Protective	CHRNB3_RORA	rs41272375_rs8029848	0.25 [0.08–0.66]	<0.01 (0.031)
	DRD4_RORA	rs916455_rs12438315	0.40 [0.21–0.77]	<0.01 (0.031)
	ZBTB20	rs113104147	0.42 [0.26–0.66]	<0.001 (<0.01)
Illicit or Recreational Drugs				
Risk	RORA_ZBTB20	rs341365_rs76374584	10.09 [2.03–72.56]	0.010 (0.028)
	ZBTB20_ZBTB20	rs73224513_rs17628822	8.85 [1.63–46.63]	<0.01 (0.028)
	RORA_ZBTB20	rs75084363_rs17628822	3.76 [1.42–10.11]	<0.01 (0.027)
Protective	PER1	rs71371830	0.39 [0.20–0.71]	<0.01 (0.024)
	RORA	rs75084363	0.43 [0.21–0.79]	0.011 (0.030)
Prescription or Over-the-Counter Medication				
Risk	RORA_RORA	rs79360097_rs8027032	8.27 [1.60–61.95]	0.017 (0.048)
	NFIL3_RORA	rs62565796_rs190776828	7.31 [1.73–29.90]	<0.01 (0.035)
	DRD4_ZBTB20	rs916455_rs11920889	6.09 [1.29–25.56]	0.016 (0.048)
Protective	RORA	rs79360097	0.17 [0.03–0.55]	0.014 (0.048)
	RORB	ch9:77273612	0.28 [0.08–0.69]	0.015 (0.048)
	DRD4	rs916455	0.40 [0.18–0.80]	0.017 (0.048)

## Data Availability

The code is available in a publicly accessible repository. Restrictions apply to the availability of these data. Data were obtained from the UK Biobank database and are available only with the permission of the UK Biobank.

## Supplementary Materials

The following supporting information can be downloaded at: https://www.mdpi.com/article/10.3390/brainsci14121282/s1. Supplementary Materials include an extended methods section, list of the genes studied, and all results (significant or otherwise) for logistic regression, mediation, and association rules analyses. References [101,102,103,104,105,106,107,108] are cited in Supplementary Materials.
