# Supplementary material for: Sex- and Substance-Specific Associations of Circadian-Related Genes with Addiction in the UK Biobank Cohort Implicate Neuroplasticity Pathways
Source: Brain Sci. 2024 Dec 20;14(12):1282. doi: 10.3390/brainsci14121282 (PMC11674644; doi:10.3390/brainsci14121282)
Supplement: Supplementary file 1 [file brainsci-14-01282-s001.zip › brainsci-3335765-supplementary.pdf]

Supplementary Materials and Methods

We followed a methodology similar to that of Minbay et al. (Minbay et al., 2024). Supplementary Figure 1 gives an overview of the methodology.

Data

Data properties, selection, and retrieval

We sourced our data for this study from the UK Biobank (UKB), a comprehensive online database storing genetic and clinical information from over 500,000 UK adults. Participants were initially recruited between 2006 and 2010, with additional enrollments over time, and provided electronic consent during recruitment. Genome-wide genotyping was conducted using the "Applied Biosystems UK BiLEVE Axiom Array" and the "UK Biobank Axiom Array." Approximately 850,000 variants were directly genotyped, with over 90 million variants imputed using the IMPUTE4 program and reference panels from the Haplotype Reference Consortium, UK10K, and 1000 Genomes phase 3 (Bycroft et al., 2018). The UKB cohort includes a higher proportion of female participants and is characterized by better average health and socioeconomic status compared to the general UK population (Fry et al., 2017). This study focused on a subset of participants who responded to a binary question about experiencing addiction or dependence on substances (excluding cigarettes and coffee) or behaviors such as gambling (UKB data field 20401; Bycroft et al., 2018).

To identify the variants of focus for our study, we conducted a literature review and searched GeneCards for terms "addiction" and "circadian," and identified 50 genes associated with circadian rhythm and/or addiction. A gene called DELEC1 also showed up in our GeneCards search, and it was also included in this study as it had been previously identified with mood disorders in a study from our lab (Minbay et al., 2024). We downloaded the imputed genotype data for all participants using UKB's gfetch program, and extracted the participants' data on the Single Nucleotide Polymorphisms (SNP) that are located on the 51 identified genes and their adjacent genomic regions (± 6000 base pairs) using the bgen Python library. In this study, we use the term "SNP" broadly to encompass all variants, not limited to single nucleotide variants. Our literature review additionally identified several confounding clinical factors (we use "clinical factors" and "confounding factors" interchangeably after this point). Four of these factors had related data fields in the UKB: sex (31), age (34), chronotype (1180), and the Townsend Deprivation Index (TSDI) (22189); the numbers in parentheses indicate the identification number of each factor's data field in the UKB (Bycroft et al., 2018). After retrieving the relevant data, participants belonging to close kinship groups were excluded using the ukbb_parser Python library (Zhu et al., 2019). Additionally, individuals with missing or indeterminate responses ("prefer not to answer" or "do not know") for any of the retrieved clinical factor fields were removed. Following these filtering steps, the final dataset comprised 98,800 participants aged 39 to 72.

From those participants (n=98,800), we identified a subset of 5366 participants who had also indicated whether they had experienced addiction or dependence specifically towards alcohol (20406), illicit or recreational drugs (e.g., narcotics, 20456), and prescription or over-the-counter medicine (e.g., opioids, 20503) through additional yes/no questions; the numbers in parentheses once again indicate the identifier of each data field. We hereby refer to the investigation on the larger group as the Study One, and the three investigations on the subset of participants as the Study Two or substudies. We also use narcotics interchangeably with illicit or recreational drugs, and opioids with prescription or over-the-counter medicine in this study for simplicity of reference; these terms are prominent examples, and not fully representative, of their respective categories.

Data pre-processing

We discretized the participants' imputed variant data (which was represented as 0 for homozygous common allele, 1 for heterozygous, and 2 for homozygous variant allele) by picking the genotype call with the highest probability. We binarized the resultant SNP values, now encoded as 0, 1, or 2 (AA, Ab, bb), using a dominant inheritance model that groups the variant and heterozygous genotypes (bb + Ab) and compares them to the common genotype (AA). For each study, we marked the participants who reported a positive answer to the respective question of the study as positive cases for that study. This resulted in 5862 cases positive for general addiction in the Study One, and for Study Two: 2246 cases for the alcohol substudy, 378 cases for the illegal drug/narcotics substudy, and 804 cases for the opioids/medicine substudy. We binarized the participants' chronotype data, which assessed individuals' diurnal preferences on a scale from 1 (definite morning type) to 4 (definite evening type), by designating individuals whose diurnal preference was 4 as cases.

Following preprocessing, we divided the Study One dataset of 98,800 individuals into male (n = 42,501) and female (n = 56,299) groups. Similarly, we split the Study Two datasets into male (n = 2,749) and female (n = 2,617) groups. We then conducted feature selection, identification of risk and protective factors, and statistical analyses separately for the male, female, and overall populations in each study. We performed mediation and association rule learning analyses only as part of the Study One and not Study Two.

Feature selection

Filtering Single Nucleotide Polymorphism (SNP) data

To narrow down the number of SNPs examined in further analyses, we used the scikit-learn and scipy Python libraries. (Pedregosa et al., 2011; Virtanen et al., 2020). To ensure robust associations, we determined participant frequency cutoffs for each group in each study (Study One male/female = 50, overall = 100; Study Two male/female = 5, overall = 10), and removed the SNPs that were found in fewer participants than the cutoff values. We performed an initial filtering of the remaining SNP data using the Chi-squared test, repeating the test across three bootstraps. Only SNPs that were consistently significant ($p < 0.05$) in all three iterations were retained, yielding approximately 250 SNPs. Next, we applied logistic multivariate regression with LASSO regularization to further refine the selection (Tibshirani, 1996). The regression analysis was

repeated for three bootstraps, with manual adjustments to the alpha regularization parameter to select approximately 50 SNPs in each iteration. SNPs selected in at least two out of three bootstraps were retained as the final set for subsequent analysis.

SNP-SNP epistatic interaction analysis

We analyzed the final set of SNPs for significant epistatic SNP-SNP interactions using the SNPAssoc R package (González et al., 2007; R Core Team, 2023). All potential confounding factors, other than sex, were included as covariates in the analysis. Interactions demonstrating significance ($p < 0.05$) were incorporated into the final feature set alongside individual SNPs and confounding factors. Interactions that did not meet the frequency thresholds (Study One: male/female = 5, overall = 10; Study Two: male/female = 2, overall = 5) were excluded.

Risk and protective factor identification

Multivariate logistic regression analysis

R was used to conduct multivariate logistic regression analysis on the final feature set. We fit an initial model using the complete final feature set. If the model didn't converge fully, we applied additional methods to eliminate collinearity: We used R to apply Cramer's V test (library: confintr) to the SNPs and SNP pairs to determine pairwise collinearity of the variables: for every variable pair that had a Cramer's V-score greater than 0.8, the variable that had a weaker odds ratio in the previous (partially converged) model was removed from the model (Cramér, 1991). We repeated this procedure of fitting a model and eliminating variables via Cramer's V test until the logistic regression model converged correctly. We also calculated the variables' GVIF scores (library: glmtoolbox) to further reduce collinearity by eliminating variables with a score greater than 5 (Fox & Monette, 1992). We trained the final logistic regression model with the remaining variables and adjusted the p-values using Benjamini-Hochberg corrections to account for multiple testing.

For each study, risk SNPs were defined as those with odds ratios (OR) greater than 1 in the final regression model based on the respective response variable of the particular study. Conversely, protective SNPs were defined as those with an OR less than 1. Using this classification, we identified the genes containing these SNPs as risk or protective factor genes. A risk or protective factor gene in each study suggests that mutations (SNPs) within that gene may have harmful or protective effects on the response variable of that study. We reported SNPs and SNP-SNP epistatic interactions that remained significant post p-value correction, and chose them for further statistical analyses.

Logistic Regression network & visualization

Based on the results of each final regression model, we constructed a network where the SNPs were represented as nodes and SNP interactions were represented as edges between their respective SNPs. We

used p-values and odds ratios of the nodes and edges to group them. We used Gephi software to visualize the resultant networks in a circular layout (Bastian et al., 2009).

Mediation Analysis

To explore mediation effects in the Study One, we used the *mediation* library in R to investigate the influence of diurnal preference on significant SNPs and SNP interactions identified through regression analysis (Tingley et al., 2014). In all analyses, we accounted for all relevant confounds, including clinical factors (other than sex), and other significant SNPs and SNP-SNP epistatic interactions.

Association Rule Learning (ARL)

We conducted association rule learning analysis on the significant SNPs and SNP interactions from Study One using the arules package in R (Hahsler et al., 2011). In this context, support refers to the likelihood of a group of factors occurring together in a dataset entry; confidence indicates the probability of observing the outcome when the group of factors co-occurs; and lift represents the ratio of the observed confidence to the expected confidence if the factors and outcome were independent. We included the significant SNP-SNP epistatic interactions as individual factors in the factor group and additionally incorporated chronotype to explore its interactions with the significant SNPs. To ensure tractability, we limited the analysis to associations with a minimum lift of 2 and a maximum factor group size of 5. Finally, we visualized the discovered associations as graphs using the igraph and legendary packages in R (Csárdi & Nepusz, 2006; Smith, 2018/2023).

Code Availability

A summary of the methods is outlined in the flowchart (Supplementary Figure 1). The source code for all procedures described above is available at: https://github.com/l1gh7vel/UK-Biobank_Addiction_Study.git.

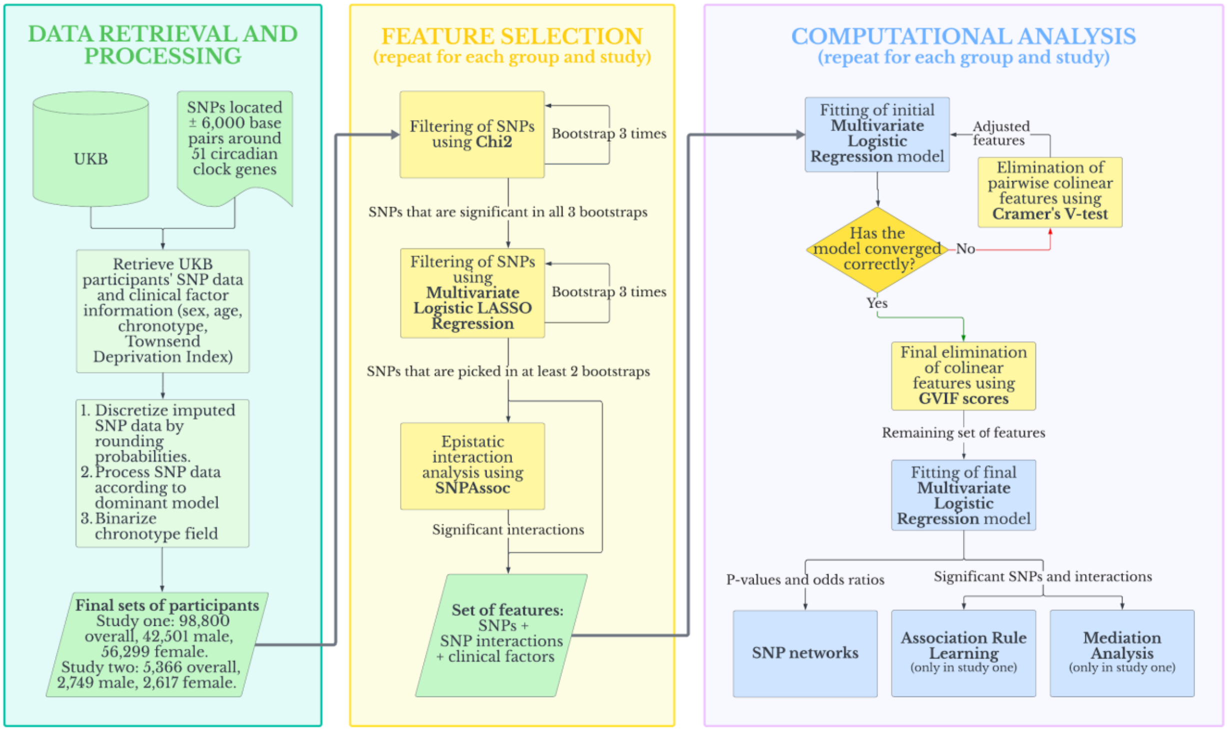

Supplementary Figure S1. Flowchart overviewing methodology. SNPs refer to all gene variants and not just single nucleotide ones. Study One refers to the general addiction study, comparing people who had any addiction to controls. Study Two comprises substudies specific to the type of substance addiction indicated by the people who were addicted in Study One (alcohol, illicit/recreational drugs, or prescription/over-the-counter medication). Extremely rare SNPs were removed if they were in fewer participants than the following frequency cutoffs: Study One male/female = 50, overall = 100; Study Two male/female = 5, overall = 10. In feature selection, the epistatic interaction analyses were controlled for all present clinical covariates.

Supplementary Table S1. Associations of circadian gene polymorphisms and clinical factors with addiction in the overall sample population. All addiction risk and protective factors in the overall UK Biobank cohort were identified using multivariate logistic regression. The results include odds ratios (OR) and 95% confidence intervals (CI). Positions for SNPs without rsIDs are given according to GRCh37.

Variable	rs #	OR [95% CI]	P value (adj)
TSDI		1.11 [1.10–1.12]	<0.001 (<0.001)
Age		0.97 [0.97–0.98]	<0.001 (<0.001)
Chronotype		1.76 [1.63–1.90]	<0.001 (<0.001)
ZBTB20	rs574306550	1.31 [1.15–1.49]	<0.001 (<0.001)
ZBTB20	rs141689778	1.30 [1.13–1.50]	<0.001 (<0.01)
RORA	ch15:61208823ATT/A	0.55 [0.40–0.74]	<0.001 (<0.01)
RORA	rs551091515	1.36 [1.15–1.60]	<0.001 (<0.01)
NPAS2	rs62156095	0.79 [0.69–0.90]	<0.001 (<0.01)
GSK3B	rs575769812	1.27 [1.11–1.45]	<0.001 (<0.01)
GABRA2	ch4:46378774TCTCCC/T	0.85 [0.78–0.93]	<0.001 (<0.01)
NPAS2	rs7587470	1.20 [1.08–1.34]	<0.001 (<0.01)
RORA	rs139781931	1.34 [1.12–1.60]	<0.01 (<0.01)
PPARGC1B	rs78814834	1.18 [1.07–1.30]	<0.01 (<0.01)
GSK3B_ZBTB20	rs138229381_ch3:114241174AG/A	1.45 [1.15–1.83]	<0.01 (<0.01)
CRY1	rs77706154	1.15 [1.05–1.26]	<0.01 (<0.01)
NPAS2	rs3811561	1.14 [1.05–1.23]	<0.01 (<0.01)
RORA	rs62006786	1.08 [1.03–1.15]	<0.01 (0.012)
HTR2A	rs80298007	0.82 [0.71–0.94]	<0.01 (0.013)
ZBTB20_PPARGC1B	rs574306550_rs17110463	0.48 [0.28–0.78]	<0.01 (0.017)
BMAL1_CRY1	rs34188368_rs77706154	0.75 [0.60–0.91]	<0.01 (0.017)
RORA_CLOCK	ch15:61208823ATT/A_rs4864991	1.79 [1.18–2.73]	<0.01 (0.018)
CLOCK_NPAS2	ch4:56314753GCGCGCGCA/G_rs3811561	1.22 [1.06–1.40]	<0.01 (0.018)
GSK3B	rs138229381	0.75 [0.60–0.92]	<0.01 (0.018)
CLOCK_GSK3B	rs4864991_rs138229381	1.37 [1.09–1.73]	<0.01 (0.020)
CLOCK	rs4864991	1.08 [1.02–1.14]	<0.01 (0.021)
DELEC1	rs13290457	0.91 [0.84–0.98]	<0.01 (0.021)
ZBTB20	rs189085886	0.72 [0.56–0.91]	<0.01 (0.021)
ZBTB20	rs116191474	0.82 [0.70–0.95]	0.010 (0.022)
NPAS2_GSK3B	rs62156095_rs138229381	1.74 [1.12–2.62]	<0.01 (0.022)
TEF	rs117971149	0.84 [0.74–0.96]	0.011 (0.023)
CSNK1E	rs9680560	0.91 [0.84–0.98]	0.012 (0.024)
HTR2A_RORA	rs80298007_rs551091515	1.97 [1.13–3.27]	0.012 (0.024)
CRY1_GSK3B	rs77706154_rs138229381	1.48 [1.08–2.00]	0.013 (0.024)
ZBTB20_DELEC1	rs574306550_rs36078239	0.68 [0.50–0.92]	0.013 (0.024)
NPAS2_ZBTB20	rs7587470_ch3:114241174AG/A	0.86 [0.76–0.97]	0.013 (0.024)
ZBTB20_ZBTB20	rs116191474_rs189085886	3.89 [1.12–10.43]	0.014 (0.026)
RORA	rs75989231	0.86 [0.76–0.97]	0.015 (0.026)
NR1D2_RORA	rs11713246_rs551091515	0.72 [0.55–0.94]	0.018 (0.031)
DELEC1	rs71505546	1.08 [1.01–1.15]	0.023 (0.037)
DELEC1_ZBTB20	rs36078239_rs116752888	1.40 [1.04–1.85]	0.023 (0.037)

NPAS2_ZBTB20	rs3811561_ch3:114241174AG/A	0.88 [0.78–0.98]	0.025 (0.040)
NR1D2_GABRA2	rs11713246_ch4:46378774TCTCCC/T	1.17 [1.02–1.35]	0.027 (0.041)
CRY1_RORA	rs77706154_rs551091515	0.63 [0.40–0.94]	0.032 (0.047)
BMAL1_RORB	rs34188368_rs11144030	0.62 [0.40–0.94]	0.032 (0.047)
RORA_RORB	rs28410611_rs11144030	0.49 [0.24–0.90]	0.033 (0.047)
GSK3B_ZBTB20	rs575769812_rs116752888	0.38 [0.13–0.85]	0.037 (0.052)
DELEC1_NPAS2	rs13290457_rs7587470	1.15 [1.01–1.31]	0.040 (0.055)
GSK3B_ZBTB20	rs575769812_rs141689778	0.39 [0.13–0.88]	0.043 (0.058)
GSK3B_NPAS2	rs334558_rs7587470	0.89 [0.79–1.00]	0.046 (0.061)
PPARGC1B_CSNK1E	rs78814834_rs9680560	0.77 [0.58–1.00]	0.051 (0.066)
NR1D2	rs11713246	1.06 [1.00–1.13]	0.056 (0.071)
NPAS2_ZBTB20	rs62156095_rs116752888	1.58 [0.96–2.48]	0.057 (0.071)
ZBTB20_CRY1	rs116191474_rs77706154	0.64 [0.39–1.01]	0.070 (0.085)
RORA_NPAS2	rs139781931_rs62156095	0.29 [0.05–0.95]	0.089 (0.107)
IL6	rs116941259	0.88 [0.75–1.03]	0.115 (0.136)
RORA	rs28410611	0.91 [0.80–1.04]	0.175 (0.203)
GSK3B	rs189174	0.96 [0.90–1.02]	0.194 (0.221)
CLOCK	ch4:56314753GCGCGCGCA/G	0.97 [0.89–1.06]	0.499 (0.559)
RORB	rs11144030	0.95 [0.80–1.12]	0.552 (0.607)
BMAL1	rs34188368	0.97 [0.88–1.08]	0.572 (0.618)
ZBTB20	rs116752888	1.04 [0.89–1.21]	0.598 (0.637)
RORA_RORA	rs28410611_rs17204496	1.03 [0.87–1.22]	0.748 (0.783)
ZBTB20_IL6	rs189085886_rs116941259	0.00 [0.00–0.00]	0.876 (0.903)
BMAL1	rs7933521	0.99 [0.91–1.09]	0.910 (0.924)
ZBTB20	ch3:114241174AG/A	1.00 [0.93–1.08]	0.940 (0.940)

Note: Table is sorted by p values.

Supplementary Table S2. Associations of circadian gene polymorphisms and clinical factors with addiction in the female sample population. All addiction risk and protective factors in the female UK Biobank cohort were identified using multivariate logistic regression). The results include odds ratios (OR) and 95% confidence intervals (CI). Positions for SNPs without rsIDs are given according to GRCh37.

Variable	rs #	OR [95% CI]	P value (adj)
TSDI		1.10 [1.10–1.12]	<0.001 (<0.001)
Age		0.97 [0.97–0.98]	<0.001 (<0.001)
Chronotype		1.62 [1.45–1.81]	<0.001 (<0.001)
VIPR2	rs185588523	1.82 [1.41–2.31]	<0.001 (<0.001)
RORA	rs576552710	1.49 [1.25–1.76]	<0.001 (<0.001)
PER3_CHRNB3	rs143075778_rs56339363	8.44 [2.53–24.25]	<0.001 (<0.01)
NPAS2	rs150840995	0.40 [0.23–0.64]	<0.001 (<0.01)
CSNK1E	rs113651082	0.82 [0.73–0.92]	<0.001 (<0.01)
RORA	rs28652652	0.58 [0.41–0.80]	<0.01 (0.011)
PER3	rs143075778	0.51 [0.33–0.76]	<0.01 (0.012)
CRY1	rs77706154	1.19 [1.06–1.33]	<0.01 (0.014)
DRD2	rs117317480	1.22 [1.07–1.37]	<0.01 (0.014)
RORA	rs146234936	1.42 [1.12–1.78]	<0.01 (0.019)
RORA_PPARGC1B	rs11071580_rs372393431	0.71 [0.56–0.89]	<0.01 (0.019)
LINC-ROR	rs117949631	1.31 [1.09–1.57]	<0.01 (0.020)
IL6_CREB1	rs73683966_rs76863021	2.32 [1.31–4.16]	<0.01 (0.021)
ZBTB20	rs17616358	0.86 [0.78–0.96]	<0.01 (0.023)
RORB	rs188874518	0.41 [0.21–0.73]	<0.01 (0.023)
ZBTB20_ZBTB20	rs880744_ch3:114241174AG/A	0.43 [0.23–0.77]	<0.01 (0.028)
CLOCK	rs113599879	0.68 [0.51–0.89]	<0.01 (0.029)
DRD2	rs117816894	0.78 [0.64–0.94]	<0.01 (0.039)
TEF_LINC-ROR	ch22:41774154AGCTTCCTT/A_rs117949631	0.52 [0.30–0.84]	0.012 (0.045)
IL6	rs73683966	0.90 [0.83–0.98]	0.013 (0.046)
ZBTB20	rs880744	0.63 [0.43–0.90]	0.013 (0.046)
CHRNB3_LINC-ROR	rs149861338_rs117949631	1.97 [1.12–3.32]	0.014 (0.046)
GSK3B_RORA	rs575769812_rs28652652	2.71 [1.18–5.81]	0.014 (0.046)
PER2_NPAS2	ch2:239179064TA/T_rs7587470	0.76 [0.61–0.95]	0.016 (0.052)
TEF_CIPC	ch22:41774154AGCTTCCTT/A_rs57864075	1.29 [1.04–1.59]	0.018 (0.054)
GSK3B_ZBTB20	rs3755557_rs73224506	1.62 [1.07–2.40]	0.019 (0.055)
DRD2_ZBTB20	rs117317480_rs880744	2.02 [1.08–3.58]	0.021 (0.059)
DRD2_RORA	rs117317480_rs576552710	0.47 [0.24–0.86]	0.023 (0.063)
CSNK1E_DRD2	rs113651082_rs117816894	1.62 [1.05–2.44]	0.023 (0.063)
GSK3B	rs3755557	0.88 [0.78–0.99]	0.028 (0.068)
CREB1	rs76863021	0.60 [0.37–0.93]	0.030 (0.068)
DELEC1	rs12337198	0.92 [0.85–0.99]	0.026 (0.068)
PER2_ZBTB20	rs76554193_ch3:114241174AG/A	0.74 [0.56–0.97]	0.029 (0.068)
TEF_ZBTB20	ch22:41774154AGCTTCCTT/A_rs17616358	1.26 [1.02–1.55]	0.030 (0.068)
CRY1_VIPR2	rs77706154_rs185588523	0.35 [0.12–0.82]	0.030 (0.068)
IL6_ZBTB20	rs73683966_rs880744	1.70 [1.06–2.73]	0.028 (0.068)
GSK3B_RORB	ch3:119673157AAACAG/A_rs188874518	2.56 [1.04–5.93]	0.032 (0.069)

CSNK1E_ZBTB20	rs113651082_rs73224506	1.61 [1.03–2.46]	0.033 (0.070)
DRD2_DELEC1	rs117317480_rs184780831	0.33 [0.10–0.81]	0.034 (0.071)
PER2	ch2:239179064TA/T	0.86 [0.75–0.99]	0.037 (0.073)
TEF_ZBTB20	ch22:41774154AGCTTCCTT/A_rs2139803	0.77 [0.60–0.98]	0.037 (0.073)
GSK3B_PPARGC1B	ch3:119793199CT/C_rs372393431	0.81 [0.67–0.99]	0.038 (0.073)
CIPC_ZBTB20	rs57864075_ch3:114241174AG/A	1.19 [1.01–1.42]	0.042 (0.079)
CLOCK_PPARGC1B	rs113599879_ch5:149183853TG/T	1.57 [1.01–2.40]	0.042 (0.079)
NPAS2	rs72816924	1.19 [1.00–1.42]	0.044 (0.079)
RORA_BMAL2	rs576552710_rs71541529	0.30 [0.07–0.83]	0.046 (0.081)
TEF	ch22:41774154AGCTTCCTT/A	0.87 [0.75–1.00]	0.051 (0.086)
CLOCK_CHRNB3	ch4:56314753GCGCGCGCA/G_rs56339363	0.43 [0.16–0.93]	0.051 (0.086)
PER2_GSK3B	ch2:239179064TA/T_ch3:119793199CT/C	1.22 [1.00–1.50]	0.051 (0.086)
ZBTB20_RORB	rs2139803_rs188874518	2.29 [0.96–5.25]	0.054 (0.086)
CLOCK_ZBTB20	rs113599879_rs2139803	1.55 [0.98–2.39]	0.054 (0.086)
PER2_ZBTB20	ch2:239179064TA/T_rs73224506	0.60 [0.34–0.98]	0.053 (0.086)
TEF_VIPR2	ch22:41774154AGCTTCCTT/A_rs185588523	0.48 [0.21–0.97]	0.057 (0.090)
NPAS2	rs7587470	1.11 [0.99–1.24]	0.062 (0.095)
ZBTB20	rs76512427_1	0.89 [0.78–1.01]	0.067 (0.101)
LINC-ROR_RORA	rs117949631_rs576552710	0.38 [0.12–0.95]	0.068 (0.102)
ZBTB20_NPAS2	rs2139803_rs72816924	0.71 [0.48–1.02]	0.070 (0.103)
DELEC1	rs184780831	0.82 [0.65–1.01]	0.075 (0.106)
RORA_RORB	rs11071580_rs188874518	0.16 [0.01–0.78]	0.075 (0.106)
RORA_GSK3B	rs11071580_ch3:119673157AAACAG/A	0.81 [0.64–1.02]	0.078 (0.107)
PER2_GSK3B	ch2:239179064TA/T_rs575769812	1.46 [0.95–2.21]	0.078 (0.107)
BMAL2	rs71541529	0.86 [0.72–1.02]	0.082 (0.109)
GSK3B_PPARGC1B	rs17204605_ch5:149183853TG/T	1.21 [0.98–1.49]	0.081 (0.109)
GSK3B	ch3:119793199CT/C	0.90 [0.80–1.02]	0.100 (0.129)
ZBTB20_CREB1	rs17616358_rs76863021	0.55 [0.26–1.08]	0.100 (0.129)
CLOCK_NPAS2	ch4:56314753GCGCGCGCA/G_rs7587470	1.17 [0.95–1.44]	0.138 (0.176)
GSK3B_RORA	ch3:119793199CT/C_rs28652652	1.45 [0.88–2.37]	0.141 (0.176)
ZBTB20	rs2139803	0.93 [0.84–1.03]	0.164 (0.203)
ZBTB20	ch3:114241174AG/A	0.93 [0.84–1.03]	0.183 (0.218)
ZBTB20_GSK3B	rs17616358_rs575769812	1.31 [0.88–1.96]	0.183 (0.218)
GSK3B_ZBTB20	rs575769812_ch3114241174AG/A	1.31 [0.88–1.94]	0.180 (0.218)
CHRNB3	rs56339363	0.84 [0.63–1.09]	0.205 (0.240)
CHRNB3	rs149861338	1.10 [0.93–1.29]	0.265 (0.306)
PPARGC1B	rs372393431	1.07 [0.94–1.22]	0.290 (0.331)
PPARGC1B	ch5:149183853TG/T	1.05 [0.95–1.16]	0.323 (0.364)
GSK3B	rs17204605	0.92 [0.79–1.08]	0.329 (0.366)
PER2	rs76554193	0.92 [0.78–1.09]	0.366 (0.402)
CIPC	rs57864075	0.95 [0.84–1.07]	0.398 (0.432)
GSK3B	rs575769812	0.88 [0.64–1.19]	0.416 (0.447)
CLOCK	ch4:56314753GCGCGCGCA/G	1.05 [0.93–1.17]	0.432 (0.457)
RORA	rs11071580	1.03 [0.92–1.15]	0.589 (0.617)
ZBTB20	rs73224506	1.06 [0.82–1.34]	0.642 (0.664)

CLOCK_CREB1	rs113599879_rs76863021	0.00 [0.00–0.01]	0.944 (0.955)
DELEC1_CREB1	rs184780831_rs76863021	0.00 [0.00–0.01]	0.943 (0.955)
VIPR2_CHRNB3	rs185588523_rs56339363	0.00 [0.00–0.31]	0.957 (0.957)

Note: Table is sorted by p values.

Supplementary Table S3. Associations of circadian gene polymorphisms and clinical factors with addiction in the male sample population. All addiction risk and protective factors in the male UK Biobank cohort were identified using multivariate logistic regression. The results include odds ratios (OR) and 95% confidence intervals (CI). Positions for SNPs without rsIDs are given according to GRCh37.

Variable	rs #	OR [95% CI]	P value (adj)
TSDI		1.12 [1.10–1.13]	<0.001 (<0.001)
Age		0.97 [0.97–0.98]	<0.001 (<0.001)
Chronotype		1.80 [1.62–2.00]	<0.001 (<0.001)
RORA	rs147861260	2.24 [1.54–3.17]	<0.001 (<0.001)
GSK3B	rs13082848	0.69 [0.58–0.83]	<0.001 (<0.001)
CSNK1E	rs113075284	0.74 [0.64–0.86]	<0.001 (<0.001)
BMAL1	rs75114867	0.73 [0.61–0.86]	<0.001 (<0.01)
CLOCK	rs4864991	1.15 [1.06–1.24]	<0.001 (<0.01)
RORA	rs62006786	1.14 [1.06–1.24]	<0.001 (<0.01)
SIRT1	rs35923230	1.45 [1.16–1.79]	<0.001 (<0.01)
VIP	rs562200953	1.70 [1.22–2.30]	<0.01 (<0.01)
NR3C1	rs10042042	0.78 [0.67–0.91]	<0.01 (0.011)
VIPR2	rs118066386	0.74 [0.61–0.89]	<0.01 (0.014)
DRD4_GSK3B	rs79177795_rs13082848	1.72 [1.19–2.44]	<0.01 (0.018)
LEP_CSNK1E	rs77947631_rs113075284	2.48 [1.29–4.52]	<0.01 (0.023)
RORB	rs11790962	1.19 [1.05–1.34]	<0.01 (0.023)
CAVIN3_CSNK1E	rs112063177_rs138711638	3.00 [1.33–6.25]	<0.01 (0.024)
HTR2A	rs33974073	0.86 [0.78–0.96]	<0.01 (0.032)
NR3C1_NPAS2	rs10042042_rs6542994	1.39 [1.09–1.78]	<0.01 (0.037)
OPN4	rs141688634	1.30 [1.05–1.61]	0.015 (0.056)
CRY1_RORB	rs117461935_rs11790962	0.52 [0.30–0.86]	0.015 (0.056)
CSNK1E_BMAL1	rs138711638_rs75114867	2.30 [1.14–4.38]	0.015 (0.056)
CRY1	rs117461935	1.20 [1.01–1.41]	0.033 (0.071)
CSNK1E	rs138711638	1.28 [1.01–1.61]	0.035 (0.071)
CSNK1D	rs141681605	0.84 [0.72–0.98]	0.026 (0.071)
RORA	rs76263497	0.91 [0.83–0.99]	0.036 (0.071)
BMAL1	rs190245148	0.76 [0.58–0.97]	0.035 (0.071)
RORA	rs551091515	1.22 [1.01–1.48]	0.039 (0.071)
GABRA2	rs4695148	1.11 [1.01–1.21]	0.028 (0.071)
NPAS2	rs151086272	1.45 [1.04–1.99]	0.024 (0.071)
CSNK1D	rs796078933	1.12 [1.00–1.25]	0.040 (0.071)
CAVIN3_RORA	rs112063177_rs74677486	0.22 [0.04–0.70]	0.035 (0.071)
NR1D1_RORA	rs117054360_rs74677486	1.92 [1.06–3.29]	0.023 (0.071)
CSNK1E_RORA	rs138711638_rs551091515	0.21 [0.03–0.74]	0.039 (0.071)
CRY1_SIRT1	rs117461935_rs35923230	0.12 [0.01–0.58]	0.040 (0.071)
BMAL1_RORB	rs75114867_rs11790962	1.53 [1.03–2.23]	0.031 (0.071)
RORA_RORA	rs147861260_rs62006786	0.44 [0.20–0.89]	0.030 (0.071)
RORA_ZBTB20	rs62006786_rs140404597	0.67 [0.47–0.94]	0.022 (0.071)
NR1D1_RORA	rs117054360_rs76263497	0.62 [0.41–0.92]	0.021 (0.071)
RORA_NPAS2	rs551091515_rs76023220	1.86 [1.05–3.16]	0.026 (0.071)

CSNK1E_HTR2A	rs113075284_rs33974073	1.41 [1.01–1.94]	0.038 (0.071)
RORA_GABRA2	rs71122899_ch4:46378774TCTCCC/T	0.78 [0.62–0.98]	0.034 (0.071)
OPN4_NPAS2	rs141688634_rs76023220	1.82 [1.01–3.17]	0.039 (0.071)
GABRA2_CLOCK	rs138396385_rs4864991	1.53 [1.04–2.27]	0.032 (0.071)
GABRA2_RORA	rs138396385_rs75627031	1.85 [1.02–3.20]	0.034 (0.071)
CRY1_LEP	rs117461935_rs77947631	2.37 [0.99–5.20]	0.039 (0.071)
RORA_GABRA2	rs75627031_ch4:46378774TCTCCC/T	1.37 [1.02–1.84]	0.036 (0.071)
CAVIN3_NPAS2	rs112063177_rs76023220	0.41 [0.16–0.89]	0.042 (0.074)
CIPC	rs79607813	0.83 [0.70–0.99]	0.043 (0.074)
HTR2A_RORA	rs17359763_rs76263497	0.68 [0.46–0.98]	0.045 (0.074)
NPAS2_NPAS2	rs13017718_rs151086272	1.90 [1.00–3.52]	0.045 (0.074)
CAVIN3_HTR2A	rs34521003_rs33974073	1.87 [0.98–3.41]	0.049 (0.079)
DRD4	rs79177795	1.11 [1.00–1.22]	0.051 (0.081)
CAVIN3_GABRA2	rs34521003_ch4:46378774TCTCCC/T	0.36 [0.11–0.89]	0.051 (0.081)
CAVIN3	rs34521003	0.73 [0.51–1.00]	0.059 (0.084)
NR1D1_CSNK1D	rs117054360_rs796078933	0.57 [0.31–0.98]	0.057 (0.084)
NPAS2_RORB	rs13017718_rs11790962	0.67 [0.44–0.99]	0.055 (0.084)
IL6_VIP	rs7802308_rs562200953	0.51 [0.24–0.99]	0.058 (0.084)
CAVIN3_GABRA2	rs112063177_ch4:46378774TCTCCC/T	0.55 [0.29–0.98]	0.056 (0.084)
CSNK1D_LEP	rs141681605_rs77947631	0.15 [0.01–0.69]	0.060 (0.084)
NPAS2_NPAS2	rs115957893_rs6542994	1.15 [0.99–1.33]	0.060 (0.084)
RORA	rs58612728	0.92 [0.84–1.00]	0.062 (0.085)
LEP	rs28954369	0.91 [0.83–1.01]	0.070 (0.095)
RORA	rs73434659	0.87 [0.74–1.02]	0.086 (0.114)
RORA_VIP	rs75627031_rs562200953	1.94 [0.86–4.11]	0.093 (0.121)
RORA_VIP	rs73434659_rs562200953	0.18 [0.01–0.91]	0.102 (0.131)
NPAS2	rs13017718	0.90 [0.78–1.02]	0.114 (0.144)
RORA	rs74677486	0.88 [0.75–1.03]	0.118 (0.147)
LEP	rs77947631	1.23 [0.94–1.57]	0.120 (0.148)
RORA_NPAS2	rs75627031_rs151086272	0.41 [0.10–1.21]	0.156 (0.189)
GABRA2	ch4:46378774TCTCCC/T	0.94 [0.83–1.06]	0.316 (0.378)
RORA	rs71122899	0.95 [0.87–1.05]	0.326 (0.384)
NPAS2	rs76023220	1.07 [0.93–1.23]	0.361 (0.420)
CAVIN3	rs112063177	0.92 [0.73–1.14]	0.450 (0.516)
IL6	rs7802308	0.97 [0.89–1.06]	0.471 (0.533)
HTR2A	rs17359763	0.95 [0.80–1.12]	0.512 (0.572)
NR1D1	rs117054360	0.94 [0.76–1.15]	0.543 (0.599)
RORA	rs75627031	1.04 [0.90–1.19]	0.586 (0.638)
ZBTB20	rs140404597	0.95 [0.76–1.16]	0.594 (0.639)
GABRA2	rs138396385	0.94 [0.70–1.24]	0.660 (0.701)
NPAS2	rs6542994	1.01 [0.91–1.11]	0.897 (0.941)
BMAL1_OPN4	rs190245148_rs141688634	-	0.954 (0.966)
HTR2A_RORA	rs17359763_rs147861260	-	0.955 (0.966)
NPAS2_NPAS2	rs151086272_rs76023220	-	0.955 (0.966)
RORA_SIRT1	rs147861260_rs35923230	-	0.971 (0.971)

Note: Table is sorted by p values.

Supplementary Table S4. Genotypic features linked to addiction mediated by chronotype in the UK Biobank cohort. Morning/evening preference data (chronotype) was used as a possible mediator of addiction in the mediation analyses performed on the overall, female, and male groups. Asterisk (*) next to the p value indicates marginally significant mediation. All covariates (SNP, SNP-SNP interaction, and clinical factors excluding sex) were added as confounds and controlled for. For SNPs without rsIDs, the GRCh37 locations are provided. No full or partial significant mediations were found for the male group.

Genotypic Feature	rs #	Mediation via Chronotype	P value
**OVERALL**			
NR1D2_RORA	rs11713246_rs551091515	-0.06	0.04
HTR2A	rs80298007	0.04	0.02
ZBTB20	rs189085886	0.04	0.02
BMAL1_RORB	rs34188368_rs11144030	-0.03	0.04
CRY1	rs77706154	0.03	0.04
NPAS2_ZBTB20	rs3811561_ch3:114241174	-0.03	0.08*
RORA_CLOCK	ch15:61208823_rs4864991	0.03	0.08*
BMAL1_CRY1	rs34188368_rs77706154	0.02	0.10*
**FEMALE**			
RORB	rs188874518	-0.02	0.10*
RORA	rs576552710	-0.02	0.10*
**MALE**			
SIRT1	rs35923230	0.05	<0.001

Supplementary Table S5. Label mappings for the SNPs used in regression, ARL, and networks. The "rs #" column represents the RSID of the variant with the GRCh37 positions provided if RSID absent, the "Gene" column lists the gene nearest to the variant, such that the variant could be either within the gene itself or up to 6,000 base pairs (6kbp) before or after it. The "Label" column is the label used to identify the SNP in the ARL and networks in the paper.

rs #	Gene	Label
rs75114867	BMAL1	BMAL1_1
rs190245148	BMAL1	BMAL1_2
rs34188368	BMAL1	BMAL1_3
rs71541529	BMAL2	BMAL2_1
rs112063177	CAVIN3	CAVIN3_1
rs34521003	CAVIN3	CAVIN3_2
rs149861338	CHRNB3	CHRNB3_1
rs56339363	CHRNB3	CHRNB3_2
rs79607813	CIPC	CIPC_1
rs57864075	CIPC	CIPC_2
rs4864991	CLOCK	CLOCK_1
rs113599879	CLOCK	CLOCK_2
ch4:56314753	CLOCK	CLOCK_3
rs76863021	CREB1	CREB1_1
rs117461935	CRY1	CRY1_1
rs77706154	CRY1	CRY1_2
rs141681605	CSNK1D	CSNK1D_1
rs796078933	CSNK1D	CSNK1D_2
rs138711638	CSNK1E	CSNK1E_1
rs113075284	CSNK1E	CSNK1E_2
rs113651082	CSNK1E	CSNK1E_3
rs9680560	CSNK1E	CSNK1E_4
rs12337198	DELEC1	DELEC1_1
rs184780831	DELEC1	DELEC1_2
rs13290457	DELEC1	DELEC1_3
rs71505546	DELEC1	DELEC1_4
rs36078239	DELEC1	DELEC1_5
rs117317480	DRD2	DRD2_1
rs117816894	DRD2	DRD2_2
rs79177795	DRD4	DRD4_1
rs4695148	GABRA2	GABRA2_1
ch4:46378774	GABRA2	GABRA2_2
rs138396385	GABRA2	GABRA2_3
rs13082848	GSK3B	GSK3B_1
rs3755557	GSK3B	GSK3B_2
ch3:119673157	GSK3B	GSK3B_3
rs575769812	GSK3B	GSK3B_4
ch3:119793199	GSK3B	GSK3B_5
rs138229381	GSK3B	GSK3B_6

rs334558	GSK3B	GSK3B_7
rs33974073	HTR2A	HTR2A_1
rs17359763	HTR2A	HTR2A_2
rs80298007	HTR2A	HTR2A_3
rs73683966	IL6	IL6_1
rs77947631	LEP	LEP_1
rs117949631	LINC-ROR	LINC-ROR_1
rs151086272	NPAS2	NPAS2_1
rs6542994	NPAS2	NPAS2_2
rs76023220	NPAS2	NPAS2_3
rs13017718	NPAS2	NPAS2_4
rs150840995	NPAS2	NPAS2_5
rs72816924	NPAS2	NPAS2_6
rs7587470	NPAS2	NPAS2_7
rs62156095	NPAS2	NPAS2_8
rs3811561	NPAS2	NPAS2_9
rs117054360	NR1D1	NR1D1_1
rs11713246	NR1D2	NR1D2_1
rs10042042	NR3C1	NR3C1_1
rs141688634	OPN4	OPN4_1
ch2:239179064	PER2	PER2_1
rs76554193	PER2	PER2_2
rs143075778	PER3	PER3_1
ch5:149183853	PPARGC1B	PPARGC1B_1
rs372393431	PPARGC1B	PPARGC1B_2
rs78814834	PPARGC1B	PPARGC1B_3
rs17110463	PPARGC1B	PPARGC1B_4
rs147861260	RORA	RORA_1
rs76263497	RORA	RORA_2
rs62006786	RORA	RORA_3
rs551091515	RORA	RORA_4
rs74677486	RORA	RORA_5
rs71122899	RORA	RORA_6
rs75627031	RORA	RORA_7
rs576552710	RORA	RORA_8
rs146234936	RORA	RORA_9
rs28652652	RORA	RORA_10
rs11071580	RORA	RORA_11
rs75989231	RORA	RORA_12
rs139781931	RORA	RORA_13
15_61208823	RORA	RORA_14
rs28410611	RORA	RORA_15
rs11790962	RORB	RORB_1
rs188874518	RORB	RORB_2
rs11144030	RORB	RORB_3

rs35923230	SIRT1	SIRT1_1
ch22:41774154	TEF	TEF_1
rs117971149	TEF	TEF_2
rs562200953	VIP	VIP_1
rs118066386	VIPR2	VIPR2_1
rs185588523	VIPR2	VIPR2_2
rs140404597	ZBTB20	ZBTB20_1
rs17616358	ZBTB20	ZBTB20_2
rs880744	ZBTB20	ZBTB20_3
ch3:114241174	ZBTB20	ZBTB20_4
rs73224506	ZBTB20	ZBTB20_5
rs2139803	ZBTB20	ZBTB20_6
rs141689778	ZBTB20	ZBTB20_7
rs116191474	ZBTB20	ZBTB20_8
rs189085886	ZBTB20	ZBTB20_9
rs574306550	ZBTB20	ZBTB20_10
rs116752888	ZBTB20	ZBTB20_11

Supplementary Table S6. Top association rules predicting addiction in the overall group with count >10. Count is defined as the number of observations where the rule and the response are observed together.

Rule	*Confidence*	*Lift*	*Count*
**{CLOCK_3, PPARGC1B_3, DELEC1_5, Chronotype}**	0.28	4.68	10
**{CLOCK_3, PPARGC1B_3, DELEC1_4, Chronotype}**	0.28	4.68	10
**{CLOCK_3, PPARGC1B_4, DELEC1_5, Chronotype}**	0.28	4.68	10
**{CLOCK_3, PPARGC1B_4, DELEC1_4, Chronotype}**	0.28	4.68	10
**{ZBTB20_10, ZBTB20_4, GSK3B_6, CLOCK_1}**	0.25	4.21	12
**{RORA_3, ZBTB20_11, NPAS2_9, Chronotype}**	0.24	4.13	12
**{ZBTB20_4, GSK3B_6, CLOCK_1, Chronotype}**	0.24	4.01	15
**{NR1D2_1, GSK3B_6, CLOCK_1, Chronotype}**	0.23	3.89	12
**{GSK3B_4, CRY1_2, Chronotype}**	0.23	3.83	10
**{GSK3B_6, CLOCK_1, NPAS2_9, Chronotype}**	0.23	3.82	12

Supplementary Table S7. Top association rules predicting addiction in the female group with count >10. Count is defined as the number of observations where the rule and the response are observed together.

Rule	*Confidence*	*Lift*	*Count*
**{ZBTB20_2, CHRNB3_1, LINC_ROR_1}**	0.2	4.04	10
**{ZBTB20_2, CHRNB3_1, Chronotype}**	0.15	2.92	13
**{GSK3B_4, ZBTB20_2, Chronotype}**	0.14	2.71	10
**{ZBTB20_4, TEF_1, CSNK1E_3, Chronotype}**	0.12	2.44	10
**{VIPR2_2, Chronotype}**	0.12	2.43	14
**{DRD2_1, RORA_11, Chronotype}**	0.12	2.35	15
**{IL6_1, CHRNB3_1, Chronotype}**	0.12	2.35	12
**{GSK3B_4, Chronotype}**	0.12	2.29	23
**{RORA_10, GSK3B_4}**	0.11	2.27	10
**{ZBTB20_4, GSK3B_4, Chronotype}**	0.11	2.27	11

Supplementary Table S8. Top association rules predicting addiction in the male group with count >10. Count is defined as the number of observations where the rule and the response are observed together.

Rule	*Confidence*	*Lift*	*Count*
**{LEP_1, RORA_3, CSNK1E_2}**	0.23	3.28	10
**{VIP_1, Chronotype}**	0.22	3.04	14
**{RORA_3, CSNK1E_2, NPAS2_2, Chronotype}**	0.21	2.92	12
**{RORA_3, NPAS2_2, RORB_1, Chronotype}**	0.2	2.78	16
**{CSNK1E_2, RORB_1, Chronotype}**	0.2	2.77	12
**{RORA_3, NPAS2_2, NR3C1_1, Chronotype}**	0.2	2.77	11
**{RORA_3, CLOCK_1, CSNK1E_2, Chronotype}**	0.19	2.62	13
**{RORA_3, CLOCK_1, SIRT1_1, NPAS2_2}**	0.17	2.46	11
**{CLOCK_1, NPAS2_2, RORB_1, Chronotype}**	0.17	2.45	12
**{HTR2A_1, RORB_1, Chronotype}**	0.17	2.4	16

Supplementary Table S9. Associations of circadian gene variants and clinical factors with alcohol addiction in the overall sample. Significant alcohol addiction risk and protective factors in the overall UK Biobank cohort were identified using multivariate logistic regression (adjusted p<0.05). The results include odds ratios (OR) and 95% confidence intervals (CI).

Variable	rs #	OR [95% CI]	P value (adj)
AANAT	rs113263038	1.76 [1.29–2.43]	<0.001 (<0.01)
RORA	rs72739551	1.52 [1.26–1.84]	<0.001 (<0.001)
ZBTB20	rs73241528	1.45 [1.12–1.87]	<0.01 (0.024)
ZBTB20	rs78803549	1.31 [1.11–1.55]	<0.01 (0.012)
CHRNB3	rs55828312	1.22 [1.07–1.40]	<0.01 (0.023)
TSDI		1.04 [0.82–1.32]	<0.001 (<0.001)
PPARGC1B	rs76861039	0.83 [0.71–0.96]	0.011 (0.047)
PPARGC1B	rs4705380	0.81 [0.71–0.92]	<0.01 (0.012)
SLC6A15	rs75139174	0.79 [0.67–0.93]	<0.01 (0.023)
RORA_DELEC1	rs12440095_rs13440344	0.61 [0.47–0.80]	<0.001 (<0.01)
DRD2_CHRNB3	rs12364283_rs55828312	0.59 [0.42–0.83]	<0.01 (0.015)

Supplementary Table S10. Associations of circadian gene variants and clinical factors with alcohol addiction in the female sample. Significant alcohol addiction risk and protective factors in the female UK Biobank cohort were identified using multivariate logistic regression (adjusted p<0.05). The results include odds ratios (OR) and 95% confidence intervals (CI).

Variable	rs #	OR[95% CI]	P value (adj)
RORA_GSK3B	rs75181035_ch3:119713907	2.85 [1.40–5.88]	<0.01 (0.029)
AANAT	rs113263038	2.83 [1.74–4.68]	<0.001 (<0.01)
CHRNB3	rs138834080	1.94 [1.24–3.04]	<0.01 (0.029)
RORA	rs551091515	1.80 [1.18–2.74]	<0.01 (0.033)
RORA	rs72739551	1.50 [1.16–1.94]	<0.01 (0.022)
GSK3B	ch3:119713907	1.42 [1.09–1.85]	<0.01 (0.042)
CSNK1D	rs7502052	1.41 [1.19–1.68]	<0.001 (<0.01)
TSDI		1.05 [0.81–1.35]	<0.001 (0.013)
DRD4	rs3758653	0.78 [0.64–0.95]	0.013 (0.049)
RORA	rs75181035	0.67 [0.49–0.91]	0.010 (0.042)
ZBTB20	rs77790747	0.58 [0.37–0.87]	<0.01 (0.042)
RORA	rs8024334	0.46 [0.26–0.79]	<0.01 (0.033)
RORA_GSK3B	rs60257905_ch3:119713907	0.32 [0.15–0.66]	<0.01 (0.022)
AANAT_RORA	rs113263038_rs75181035	0.16 [0.03–0.59]	0.011 (0.042)

Supplementary Table S11. Associations of circadian gene variants and clinical factors with alcohol addiction in the male sample. Significant alcohol addiction risk and protective factors in the male UK Biobank cohort were identified using multivariate logistic regression (adjusted p<0.05). The results include odds ratios (OR) and 95% confidence intervals (CI).

**Variable**	**rs #**	**OR [95% CI]**	**P value (adj)**
CHRNB3_ZBTB20	rs55828312_rs113104147	3.19 [1.54–6.72]	<0.01 (0.018)
CHRNB3	rs41272375	2.35 [1.62–3.46]	<0.001 (<0.001)
DRD4	rs916455	1.84 [1.25–2.73]	<0.01 (0.018)
RORA	rs12438315	1.28 [1.07–1.53]	<0.01 (0.031)
CHRNB3	rs55828312	1.24 [1.04–1.47]	0.014 (0.041)
TSDI		1.04 [0.83–1.30]	<0.01 (0.018)
BMAL2	rs192666409	0.75 [0.61–0.93]	<0.01 (0.034)
RORA	rs140033321	0.73 [0.57–0.92]	<0.01 (0.034)
CRY1	rs151322647	0.64 [0.46–0.90]	<0.01 (0.034)
ZBTB20	rs73238057	0.58 [0.38–0.88]	0.011 (0.036)
ZBTB20	rs113104147	0.42 [0.26–0.66]	<0.001 (<0.01)
DRD4_RORA	rs916455_rs12438315	0.40 [0.21–0.77]	<0.01 (0.031)
CHRNB3_RORA	rs41272375_rs8029848	0.25 [0.08–0.66]	<0.01 (0.031)

Supplementary Table S12. Associations of circadian gene variants and clinical factors with illicit or recreational drugs addiction in the overall sample. Significant risk and protective factors in the overall UK Biobank cohort were identified using multivariate logistic regression (adjusted p<0.05). The results include odds ratios (OR) and 95% confidence intervals (CI).

Variable	rs #	OR [95% CI]	P value (adj)
ZBTB20_OPN4	rs71616165_ch10:88408870	2.76 [1.26–6.14]	0.012 (0.035)
DELEC1_RORA	ch10:88408870	2.34 [1.10–4.85]	0.024 (0.049)
ZBTB20_PPARGC1B	rs71616165_rs34788898	2.16 [1.18–3.98]	0.013 (0.035)
RORA	rs28516888	2.11 [1.28–3.36]	<0.01 (0.015)
PER2	rs112007104	1.88 [1.08–3.14]	0.019 (0.043)
CRH	ch8:67084302	1.61 [1.13–2.25]	<0.01 (0.024)
DRD2	rs77541954	1.60 [1.16–2.17]	<0.01 (0.016)
ZBTB20	rs74465971	1.57 [1.11–2.17]	<0.01 (0.028)
RORA	ch3:114085720	1.52 [1.17–1.97]	<0.01 (0.015)
ZBTB20	ch15:60805890	1.51 [1.07–2.09]	0.015 (0.038)
TSDI		1.17 [0.23–6.09]	<0.001 (<0.001)
Age		0.92 [0.91–0.92]	<0.001 (<0.001)
BMAL1	rs12795264	1.46 [1.05–1.98]	0.020 (0.043)
PER3	rs1989147	0.63 [0.46–0.85]	<0.01 (0.015)
DELEC1	ch8:67084302	0.60 [0.43–0.83]	<0.01 (0.015)
RORA	rs8038077	0.56 [0.37–0.82]	<0.01 (0.018)
BMAL1_RORA	rs12363415_rs79767706	0.44 [0.21–0.87]	0.022 (0.047)
RORA_PPARGC1B	rs17303202_rs34788898	0.43 [0.23–0.80]	<0.01 (0.028)
OPN4	ch10:88408870	0.42 [0.23–0.72]	<0.01 (0.015)
ZBTB20	rs71616165_ch10:88408870	0.35 [0.13–0.75]	0.016 (0.038)

Supplementary Table S13. Associations of circadian gene variants and clinical factors with illicit or recreational drugs addiction in the female sample. Significant risk and protective factors in the female UK Biobank cohort were identified using multivariate logistic regression (adjusted p<0.05). The results include odds ratios (OR) and 95% confidence intervals (CI).

Variable	rs #	OR [95% CI]	P value (adj)
VIPR2_ZBTB20	rs183433583_rs77359140	839.72 [19.81–40471.15]	<0.001 (<0.01)
DELEC1_BMAL1	rs41278693_rs34188368	34.18 [3.16–841.14]	<0.01 (0.029)
CSNK1D_BMAL1	rs76390553_rs34188368	16.24 [2.06–149.24]	<0.01 (0.032)
USP46	rs181507842	7.50 [2.99–18.12]	<0.001 (<0.001)
ZBTB20	rs116123363	4.40 [1.75–10.19]	<0.001 (<0.01)
BMAL2	rs66669521	1.85 [1.23–2.77]	<0.01 (0.013)
TSDI		1.20 [0.12–11.70]	<0.001 (<0.001)
Age		0.91 [0.91–0.91]	<0.001 (<0.001)
HTR2A	rs9534512	0.48 [0.32–0.73]	<0.001 (<0.01)
DELEC1	rs2992140	0.39 [0.19–0.75]	<0.01 (0.029)
RORA	ch15:61377024	0.39 [0.24–0.61]	<0.001 (<0.001)
ZBTB20	rs77359140	0.11 [0.02–0.42]	<0.01 (0.026)

Supplementary Table S14. Associations of circadian gene variants and clinical factors with illicit or recreational drugs addiction in the male sample. Significant risk and protective factors in the male UK Biobank cohort were identified using multivariate logistic regression (adjusted p<0.05). The results include odds ratios (OR) and 95% confidence intervals (CI).

Variable	rs #	OR [95% CI]	P value (adj)
RORA_ZBTB20	rs341365_rs76374584	10.09 [2.03–72.56]	0.010 (0.028)
ZBTB20_ZBTB20	rs73224513_rs17628822	8.85 [1.63–46.63]	<0.01 (0.028)
RORA_ZBTB20	rs75084363_rs17628822	3.76 [1.42–10.11]	<0.01 (0.027)
RORA	rs117267768	1.93 [1.18–3.07]	<0.01 (0.027)
ZBTB20	rs78103331	1.83 [1.16–2.81]	<0.01 (0.027)
RORA	rs11634887	1.66 [1.19–2.31]	<0.01 (0.020)
RORA	rs67629419	1.55 [1.12–2.14]	<0.01 (0.027)
RORA	rs341365	1.50 [1.08–2.12]	0.018 (0.043)
TSDI		1.13 [0.43–2.98]	<0.001 (<0.001)
Age		0.91 [0.91–0.92]	<0.001 (<0.001)
RORA	rs75084363	0.43 [0.21–0.79]	0.011 (0.030)
PER1	rs71371830	0.39 [0.20–0.71]	<0.01 (0.024)

Supplementary Table S15. Associations of circadian gene variants and clinical factors with prescription or over-the-counter medication (opioid) addiction in the overall sample. Significant opioid risk and protective factors in the overall UK Biobank cohort were identified using multivariate logistic regression (adjusted p<0.05). The results include odds ratios (OR) and 95% confidence intervals (CI).

Variable	rs#	OR [95% CI]	P value (adj)
DELEC1_DRD2	rs7044600_rs61905363	2.57 [1.22–5.22]	0.011 (0.037)
RORB_ZBTB20	rs7357785_rs752404623	2.37 [1.24–4.72]	0.011 (0.037)
CSNK1E	rs113908050	2.36 [1.58–3.48]	<0.001 (<0.001)
GSK3B	rs76870804	1.82 [1.23–2.65]	<0.01 (0.015)
ZBTB20	rs115749706	1.54 [1.14–2.07]	<0.01 (0.024)
Age		1.02 [0.90–1.17]	<0.001 (<0.001)
ZBTB20	rs752404623	0.76 [0.65–0.90]	<0.01 (0.015)
DRD2	rs77655590	0.64 [0.47–0.85]	<0.01 (0.022)
CHRNB3	rs77640401	0.59 [0.40–0.85]	<0.01 (0.028)
CRY1_RORA	ch12:107430363_rs6494219	0.56 [0.35–0.89]	0.014 (0.044)
CSNK1E_ZBTB20	ch12:107430363	0.50 [0.30–0.84]	<0.01 (0.037)

Supplementary Table S16. Associations of circadian gene variants and clinical factors with prescription or over-the-counter medication (opioid) addiction in the female sample. Significant opioid risk and protective factors in the female UK Biobank cohort were identified using multivariate logistic regression (adjusted p<0.05). The results include odds ratios (OR) and 95% confidence intervals (CI).

Variable	rs #	OR [95% CI]	P value (adj)
SIRT1_RORA	rs113693349_rs8042801	37.76 [3.82–623.79]	<0.01 (0.022)
PER2_NR1D2	rs62194938_rs149810501	19.27 [2.39–184.23]	<0.01 (0.027)
CSNK1D_NPAS2	rs117549365_rs12712082	4.05 [1.61–10.45]	<0.01 (0.021)
AANAT	rs73996395	2.89 [1.83–4.53]	<0.001 (<0.001)
ZBTB20	rs10934281	1.81 [1.21–2.67]	<0.01 (0.021)
RORA	rs150656684	1.72 [1.13–2.55]	<0.01 (0.037)
TEF	rs17365991	1.61 [1.19–2.16]	<0.01 (0.020)
NPAS2	rs12712082	1.52 [1.11–2.06]	<0.01 (0.037)
PPARGC1B	rs32582	1.41 [1.08–1.84]	0.012 (0.042)
Age		1.02 [0.88–1.20]	<0.001 (<0.001)
PER1	rs3027191	0.37 [0.16–0.75]	0.011 (0.040)
PER2	rs62194938	0.34 [0.16–0.65]	<0.01 (0.021)

Supplementary Table S17. Associations of circadian gene variants and clinical factors with prescription or over-the-counter medication (opioid) addiction in the male sample. Significant opioid risk and protective factors in the male UK Biobank cohort were identified using multivariate logistic regression (adjusted p<0.05). The results include odds ratios (OR) and 95% confidence intervals (CI)..

Variable	rs #	OR [95% CI]	P value (adj)
RORA_RORA	rs79360097_rs8027032	8.27 [1.60–61.95]	0.017 (0.048)
NFIL3_RORA	rs62565796_rs190776828	7.31 [1.73–29.90]	<0.01 (0.035)
DRD4_ZBTB20	rs916455_rs11920889	6.09 [1.29–25.56]	0.016 (0.048)
CRY1_RORA	rs148619638_rs8027032	5.06 [1.41–18.97]	0.013 (0.048)
RORB_BHLHE41	rs34177511_rs117366091	4.88 [1.50–16.55]	<0.01 (0.048)
DELEC1	rs190080439	3.51 [1.57–7.41]	<0.01 (0.015)
CSNK1E	rs113908050	2.51 [1.63–3.79]	<0.001 (<0.001)
BMAL2	rs144343059	2.39 [1.19–4.50]	<0.01 (0.048)
RORB	rs138008178	2.34 [1.15–4.48]	0.014 (0.048)
NFIL3	rs62565796	2.04 [1.29–3.16]	<0.01 (0.015)
RORA	rs8027032	0.57 [0.43–0.75]	<0.001 (<0.001)
RORA	rs56219809	0.42 [0.23–0.70]	<0.01 (0.015)
DRD4	rs916455	0.40 [0.18–0.80]	0.017 (0.048)
RORB	ch9:77273612	0.28 [0.08–0.69]	0.015 (0.048)
RORA	rs79360097	0.17 [0.03–0.55]	0.014 (0.048)

Supplementary Table S18. 51 genes linked to circadian rhythm and/or addiction identified from literature search.

Core circadian genes	Circadian related genes
BHLHE40 / BHLHB2	AANAT
BHLHE41	ANNK1
ARNTL1 / BMAL1	ARNTL2 / BMAL2
CLOCK	CAVIN3
CRY1	CHRNB3
CRY2	CIART
DBP	CIPC
FBXL3	CREB1
NFIL3	CRH
NR1D1	CSNK1D
NR1D2	CSNK1E
PER1	DELEC1*
PER2	DRD2
PER3	DRD4
RORA	GABRA2
RORB	GSK3B
TEF	HES7
	HTR2A
	IL6
	LEP
	LINC-ROR
	NOCT
	NPAS2
	NR3C1
	OPN4
	PPARGC1B
	SIRT1
	SLC6A15
	TIMELESS
	TIPIN (TIMELESS Interacting Protein)
	USP46
	VIP
	VIPR2
	ZBTB20

Notes: The gene list is curated by performing a literature search and looking up genes from GeneCards as mentioned in methods, and the core-clock genes are identified using literature search (Cox & Takahashi, 2019; Korenčič et al., 2014; Stelzer et al., 2016). DELEC1 is not circadian related, but was a part of the GeneCards search for circadian genes, and was significantly associated with addiction in our results, as well as with mood disorders in our previous study (Minbay et al., 2024) so we decided to keep it.

Supplementary References

Bastian, M., Heymann, S., & Jacomy, M. (2009). Gephi: An Open Source Software for Exploring and Manipulating Networks. *Proceedings of the International AAAI Conference on Web and Social Media*, *3*(1), Article 1. https://doi.org/10.1609/icwsm.v3i1.13937

Bycroft, C., Freeman, C., Petkova, D., Band, G., Elliott, L. T., Sharp, K., Motyer, A., Vukcevic, D., Delaneau, O., O'Connell, J., Cortes, A., Welsh, S., Young, A., Effingham, M., McVean, G., Leslie, S., Allen, N., Donnelly, P., & Marchini, J. (2018). The UK Biobank resource with deep phenotyping and genomic data. *Nature*, *562*(7726), Article 7726. https://doi.org/10.1038/s41586-018-0579-z

Cox, K. H., & Takahashi, J. S. (2019). Circadian Clock Genes and the Transcriptional Architecture of the Clock Mechanism. *Journal of Molecular Endocrinology*, *63*(4), R93. https://doi.org/10.1530/JME-19-0153

Cramér, H. (1991). *Mathematical methods of statistics*. Princeton university press.

Csárdi, G., & Nepusz, T. (2006). *The igraph software package for complex network research*. https://www.semanticscholar.org/paper/The-igraph-software-package-for-complex-network-Cs%C3%A1rdi-Nepusz/1d2744b83519657f5f2610698a8ddd177ced4f5c

Fox, J., & Monette, G. (1992). Generalized Collinearity Diagnostics. *Journal of the American Statistical Association*, *87*(417), 178–183. https://doi.org/10.1080/01621459.1992.10475190

Fry, A., Littlejohns, T. J., Sudlow, C., Doherty, N., Adamska, L., Sprosen, T., Collins, R., & Allen, N. E. (2017). Comparison of Sociodemographic and Health-Related Characteristics of UK Biobank Participants With Those of the General Population. *American Journal of Epidemiology*, *186*(9), 1026–1034. https://doi.org/10.1093/aje/kwx246

González, J. R., Armengol, L., Solé, X., Guinó, E., Mercader, J. M., Estivill, X., & Moreno, V. (2007). SNPassoc: An R package to perform whole genome association studies. *Bioinformatics*, *23*(5), 654–655. https://doi.org/10.1093/bioinformatics/btm025

Hahsler, M., Chelluboina, S., Hornik, K., & Buchta, C. (2011). The arules R-Package Ecosystem: Analyzing

Interesting Patterns from Large Transaction Data Sets. *Journal of Machine Learning Research*, *12*(57), 2021–2025.

Korenčič, A., Košir, R., Bordyugov, G., Lehmann, R., Rozman, D., & Herzel, H. (2014). Timing of circadian genes in mammalian tissues. *Scientific Reports*, *4*(1), 5782. https://doi.org/10.1038/srep05782

Minbay, M., Khan, A., Ghasemi, A. R., Ingram, K. K., & Ay, A. A. (2024). Sex-specific associations between circadian-related genes and depression in UK Biobank participants highlight links to glucose metabolism, inflammation and neuroplasticity pathways. *Psychiatry Research*, *337*, 115948. https://doi.org/10.1016/j.psychres.2024.115948

Pedregosa, F., Varoquaux, G., Gramfort, A., Michel, V., Thirion, B., Grisel, O., Blondel, M., Prettenhofer, P., Weiss, R., & Dubourg, V. (2011). Scikit-learn: Machine learning in Python. *The Journal of Machine Learning Research*, *12*, 2825–2830.

R Core Team. (2023). *R: A Language and Environment for Statistical Computing*. R Foundation for Statistical Computing. https://www.R-project.org/

Smith, A. B. (2023). *Adamlilith/legendary* [R]. GitHub. https://github.com/adamlilith/legendary (Original work published 2018)

Stelzer, G., Rosen, N., Plaschkes, I., Zimmerman, S., Twik, M., Fishilevich, S., Stein, T. I., Nudel, R., Lieder, I., Mazor, Y., Kaplan, S., Dahary, D., Warshawsky, D., Guan-Golan, Y., Kohn, A., Rappaport, N., Safran, M., & Lancet, D. (2016). The GeneCards Suite: From Gene Data Mining to Disease Genome Sequence Analyses. *Current Protocols in Bioinformatics*, *54*, 1.30.1-1.30.33. https://doi.org/10.1002/cpbi.5

Tibshirani, R. (1996). Regression Shrinkage and Selection via the Lasso. *Journal of the Royal Statistical Society. Series B (Methodological)*, *58*(1), 267–288. JSTOR.

Tingley, D., Yamamoto, T., Hirose, K., Keele, L., & Imai, K. (2014). mediation: R Package for Causal Mediation Analysis. *Journal of Statistical Software*, *59*(5), 1–38. https://doi.org/10.18637/jss.v059.i05

Virtanen, P., Gommers, R., Oliphant, T. E., Haberland, M., Reddy, T., Cournapeau, D., Burovski, E., Peterson, P., Weckesser, W., Bright, J., van der Walt, S. J., Brett, M., Wilson, J., Millman, K. J.,

Mayorov, N., Nelson, A. R. J., Jones, E., Kern, R., Larson, E., … van Mulbregt, P. (2020). SciPy 1.0: Fundamental algorithms for scientific computing in Python. *Nature Methods*, *17*(3), Article 3. https://doi.org/10.1038/s41592-019-0686-2

Zhu, A., Salminen, L. E., Thompson, P. M., & Jahanshad, N. (2019). The UK Biobank Data Parser: A tool with built in and customizable filters for brain studies. *Organization for Human Brain Mapping Rome, Italy*, *6*, 9–13.
